# Effect of non-surgical periodontal therapy on risk markers of cardiovascular disease: a systematic review and meta-analysis

**DOI:** 10.1186/s12903-024-04433-0

**Published:** 2024-06-14

**Authors:** Rijing Meng, Jialei Xu, Chenrui Fan, Haiqing Liao, Zeni Wu, Qixin Zeng

**Affiliations:** 1 Department of Periodontics and Oral Medicine, College & Hospital of Stomatology, Guangxi Medical University, No. 22, Shuangyong Road, Qingxiu District, Nanning, 530021 Guangxi China; 2 Guangxi Key Laboratory of Oral and Maxillofacial Rehabilitation and Reconstruction, Nanning, China; 3 Guangxi Health Commission Key laboratory of prevention and treatment for oral infectious diseases, Nanning, China; 4 School of Population Medicine and Public Health, Chinese Academy of Medical Sciences, Peking Union Medical College, Beijing, 100730 China

**Keywords:** Cardiovascular disease, Periodontitis, Non-surgical periodontal therapy, Meta-analysis

## Abstract

**Background:**

Cardiovascular disease (CVD) is the leading cause of mortality in the world. Patients with periodontitis have a higher risk of CVD, although a causal relationship between these conditions remains unclear. Non-surgical periodontal therapy (NSPT) is able to control inflammation at local and systemic levels. This study aimed to analyze the effect of NSPT on CVD risk markers.

**Methods:**

Four electronic databases were searched from their inception to April 1, 2023, to identify and select articles without any language restrictions. Eleven CVD-related markers (e.g., C-reactive protein [CRP], Interleukin-6 [IL-6]) were selected. Meta-analyses were performed using random and fixed effect models. The differences were expressed as weighted mean differences (WMD) and 95% confidence interval (95% CI).

**Results:**

From 1353 studies, twenty-one randomized controlled clinical trials were included in the meta-analysis. Results showed a significant decrease in CRP, IL-6, and systolic blood pressure (SBP) after NSPT.

**Conclusion:**

Moderate certainty evidence shows that NSPT has a positive effect on the reduction of IL-6 and SBP in patients with periodontitis, while low certainty evidence shows that NSPT is effective for reduction of CRP. Moderate certainty evidence showed that NSPT did not show a positive effect on low-density lipoprotein (LDL), high-density lipoprotein (HDL), total cholesterol (TC) and triglycerides (TG), and low certainty evidence showed that NSPT did not show a positive effect on Interleukin-1β (IL-1β), tumor necrosis factor-alpha (TNF-α), diastolic blood pressure (DBP), and flow-mediated dilatation (FMD).

**Protocol Registration:**

The protocol was registered in the PROSPERO (International Prospective Register of Systematic Reviews), number CRD42022377565.

**Supplementary Information:**

The online version contains supplementary material available at 10.1186/s12903-024-04433-0.

## Introduction

Periodontitis is a chronic multifactorial inflammatory disease associated with dysbiotic plaque biofilms and characterized by progressive destruction of the tooth-supporting apparatus [1]. In its severe form, periodontitis is the sixth most prevalent condition in the world and afflicts about 10% of the adult population [2, 3]. Accumulating evidence has shown that untreated periodontitis is associated with several systemic conditions such as cardiovascular disease (CVD) [4], diabetes [5], chronic obstructive pulmonary disease [6], and renal diseases [7]. One possible mechanism contributing to the relationship between comorbidities and periodontitis is the low-grade systemic inflammation caused by periodontitis, a common denominator of many chronic illnesses [8]. Periodontitis is a local inflammation and a source of low-grade chronic inflammation, which results from haematogenous dissemination of periodontal bacteria or spillover of inflammatory mediators from periodontal tissues to the bloodstream [4, 9], thus contributing to the systemic inflammatory burden, increasing the risk of several chronic diseases like CVD, type II diabetes mellitus (T2DM), renal diseases, cancer and neurodegenerative disorders [10].

CVD is a broad category that includes many different pathologies, including ischemic heart disease, stroke, hypertension, rheumatic heart disease, cardio-myopathies, and atrial fibrillation [11]. It has been proved that there is an association between periodontitis and CVD, and this relation has been reported to be independent of other risk factors such as age, gender, dyslipidemia, hypertension, diabetes, and lifestyle factors such as smoking [12, 13]. Patients with severe periodontitis had higher blood neutrophil counts and pro-inflammatory mediator levels (such as IL-1, IL-6, CRP, and fibrinogen) compared to healthy controls [14–17], which were associated with a higher cardiovascular risk [18]. Furthermore, several studies indicate that periodontitis patients have a more atherogenic lipid profile in the form of elevated LDL, TG, small dense LDL, as well as very low-density lipoprotein cholesterol (VLDL), along with decreased levels of HDL concentration [17, 19]. Additionally, there is evidence from various studies that individuals with periodontitis have a higher prevalence of subclinical CVD, which is characterized by significant arterial stiffness (assessed by pulse wave velocity [PWV]) [20] and endothelial dysfunction [21]. Recent research also found a relationship between periodontitis and hypertension [22, 23].

Periodontal therapy is a standard therapeutic modality that disrupts the biofilm to control inflammation in periodontal diseases [24]. Recently, the European Federation of Periodontology (EFP) developed a S3 level clinical practice guidelines using a pre-established stepwise approach for the treatment of Stages I–III periodontitis [25]. The first step focuses on behavioural changes by motivating the patient to remove supragingival dental biofilm and control risk factors (such as smoking). Subgingival instrumentation is the main component of the second step, which may be supplemented with adjunctive therapies. If the aims of periodontal therapy have not been achieved with this sequence of steps1 and 2, the third step of repeated subgingival instrumentation or periodontal surgery is need. Non-surgical periodontal therapy (NSPT) primarily consists of subgingival scaling and root surface debridement to eliminate calculus and supra/subgingival biofilm [26], which is the gold-standard treatment for Stages I–III periodontitis [25]. A recent consensus report of the EFP and the World Heart Federation (WHF) summarized the evidence on periodontal therapy’ s effect on surrogate CVD markers [16]. The authors concluded that there is moderate evidence for reduction of low-grade inflammation as assessed by serum levels of CRP and IL-6 and improvement in surrogate measures of endothelial function (assessed by FMD).

Considering inter-trial differences, we performed an up-to-date systematic review and meta-analysis of randomized controlled clinical trials to test the hypothesis that whether NSPT, including mechanical debridement, is able to decrease CVD-related risk markers levels in patients with periodontitis compared with untreated patients.

## Methods

### Protocol registration

This systematic review was conducted according to the guideline of the PRISMA (Preferred Reporting Items for Systematic review and Meta-Analyses) (Appendix 1) and registered with the ID CRD42022377565 in the PROSPERO.

The investigation question was designed using the PICOS acronym: Population (P): patients with periodontitis; Intervention (I): Non-surgical periodontal therapy with both supra and subgingival instrumentation; Comparison (C): No treatment or oral hygiene instructions (OHI) only or control periodontal therapy (CPT, including supragingival scaling only or supragingival scaling with OHI); Outcome (O): Systemic inflammation markers: CRP, IL-6, IL-1β, TNF-α; Lipid metabolism markers: TC, TG, LDL, HDL; Vascular function indexes: SBP, DBP, FMD; Study (S): Randomized clinical trials (RCTs).

### Inclusion and exclusion criteria

Inclusion criteria were as follows: (1) RCTs that aimed to test the effects of NSPT on risk markers for cardiovascular disease; (2) studies involving periodontitis with a clear definition of periodontitis based on clinical and/or radiographic measurements of periodontal conditions, for example, periodontal probing depth (PPD), clinical attachment level (CAL) or alveolar bone height; (3) patients in intervention group undergoing NSPT in the form of OHI, supra-gingival debridement, subgingival scaling and root surface debridement with or without the use of adjunctive antiseptic and/or antibiotics, compared with patients in control group receiving no treatment or OHI only or control periodontal therapy (CPT, including supragingival scaling only or supragingival scaling with OHI); (4) studies reporting risk markers of cardiovascular disease as outcomes including (a) systemic inflammation markers: CRP, IL-6, IL-1β, TNF-α; (b) lipid metabolism markers: TC, TG, LDL, HDL; (c) vascular function indexes: SBP, DBP and FMD.

Exclusion criteria included the following: (1) types of articles with study design other than RCTs (controlled clinical trials, cohort studies, pilot studies, case control studies, review, animal studies.); (2) studies in which patients receive surgical periodontal therapy or laser therapy; (3) studies with unavailable data.

### Search methods for identification of the studies

Four electronic databases (including the National Library of PubMed, Embase, the Cochrane Library and Web of Science) were searched up from their inception to April 1, 2023. Reference lists from the included studies were searched to retrieve relevant studies not identified through other search methods. We did not use any language-specific search restrictions. Supplemental Table S1 provides a comprehensive search strategy with relevant search terms for each database.

### Study selection and data extraction

#### Study selection

Two independent reviewers selected the study according to the inclusion/exclusion criteria. Initially, search results were screened by titles and abstracts. To further evaluate eligibility, full texts of possible eligible studies were retrieved and assessed by both reviewers. Any differences of opinion about the inclusion of studies were discussed to get to a consensus, and a third reviewer arbitrated if needed.

#### Data extraction

Two reviewers carried out the data extraction independently. Disagreements were resolved through discussion, and when the dispute remained unresolved, an arbitrator was called in. Great effort was devoted in contacting authors to retrieve any missing data. The following data were collected.

- Study characteristics: author’s name, year of publication, country, study design, sample size, follow-up time.
- Participants: age, smoking status, heath status, diagnostic criteria for periodontitis.
- Interventions: intervention details including NSPT and control periodontal treatment.
- Outcomes: systemic inflammation markers: CRP, IL-6, IL-1β, TNF-α; lipid metabolism markers: TC, TG, LDL, HDL; vascular function indexes: SBP, DBP and FMD.

#### Risk of the bias

Quality assessment of all included studies was undertaken independently by two reviewers as part of the data extraction process. Bias was assessed using the RCTs risk assessment tool recommended by the Cochrane Reviewers’ Handbook-RoB 2.0 [27]. Bias assessment involved randomization process, deviations from the intended interventions, missing outcome data, measurement of the outcome, selection of the reported result.

#### Data analysis and synthesis results

Means and standard deviations (SD) were used to estimate the weighted mean difference (WMD) and its 95% confidence interval (95% CI). A *P* value less than 0.05 was considered to be significant for analysis. All statistical analyses included in the Mata-analysis used R 4.2.2 with the package “meta”. The *I*^*2*^ test was used to assess the heterogeneity of studies. *I*^*2*^ > 50% and > 75% indicated moderate heterogeneity and large heterogeneity, respectively. The fixed-effects model was used for analysis if there was no heterogeneity between the trials (*P* > 0.10, *I*^*2*^ ≤ 50%), and the random effects model was applied if there was heterogeneity (*P* < 0.10, *I*^*2*^ ≥ 50%) [28, 29]. To explore potential sources of heterogeneity in the analysis of the coprimary outcomes, subgroup analyses were conducted by systemic health status, use of adjunctive antiseptic and/or antibiotics, and follow-up time. To evaluate the robustness of pooled results, we performed sensitivity analyses by excluding studies one by one or excluding the studies in which the control group performed CPT. Egger’s test and a visual appraisal of funnel plot asymmetry were used to evaluate publication bias when at least ten RCTs were available for meta-analysis [30, 31].

### Certainty assessment

The Grading of Recommendations Assessment, Development and Evaluation (GRADE) [32] was followed to obtain the level of certainty in the body of evidence for direct estimates. The assessment encompasses the examination of the potential within-study risk of bias, directness of evidence, heterogeneity, the precision of effect estimates, and risk of publication bias. For each outcome, the level of certainty was rated as high, moderate, low, or very low.

## Results

### Search results

**Fig. 1:**
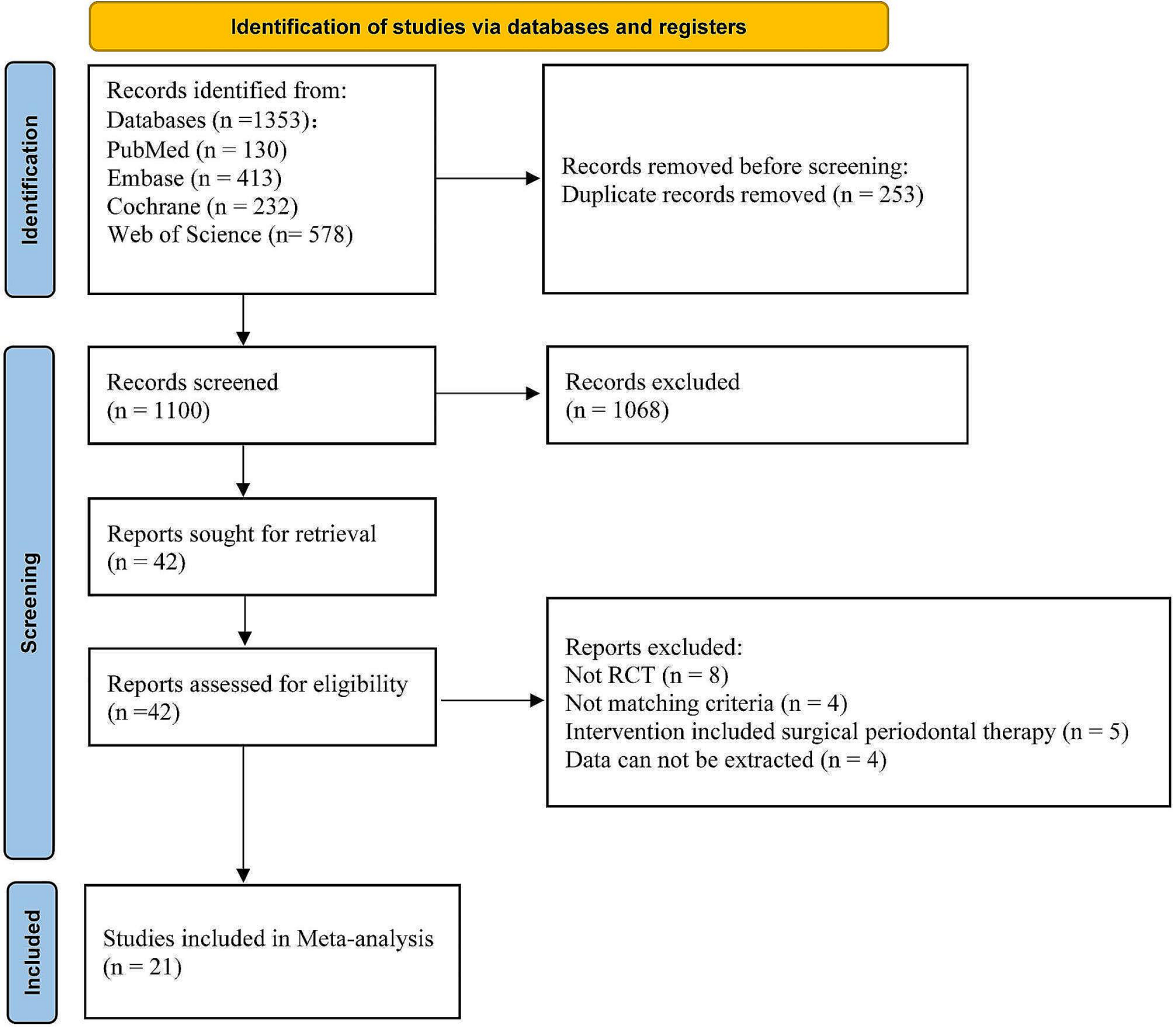
PRISMA flow diagram

The PRISMA flow diagram is shown in Fig. 1. A total of 1353 records were identified from initial search, and 253 duplicate records were removed. By screening the title and abstract, 1068 studies were excluded since they did not meet the inclusion criteria. After full text review, another 21 studies [33–53] were excluded. The list of excluded studies is provided in Supplemental Table S2. Finally, twenty-one [54–74] studies were included for further analysis.

### Study characteristics

The characteristics of the Twenty-one studies are described in Table 1. Included studies were published between 2003 and 2021 from 10 different countries. Of the 21 included studies, 6 studies were from China, 3 studies were from UK, 3 studies were from Brazil, 2 studies were from Japan, 2 studies were from Australia, and the remaining 5 were each from Jordan, Pakistan, America, Poland and Spain. The mean age of participants ranged from 38.38 ± 9.31 to 64.00 ± 14.00 years old. Current smokers were included in thirteen studies, and four studies did not report smoking status.

**Table 1:** Characteristics of the studies included in the meta-analysis

Study	Country	Healthy status	Group	Sample	Age (years) Mean ± SD	Male	BMI (Kg/m^2^) Mean ± SD	Smokers	Procedure	Follow-up
Ide 2003	UK	periodontitis	Intervention group	24	47.8 ± 7.5	13	NI	Current − 0 Former − 10 Never- 14	NSPT	3 months
			Control group	15	46.0 ± 6.2	10	NI	Current − 0 Former − 8 Never − 7	no treatment	
D’Aiuto 2005	UK	periodontitis	Intervention group-1	20	49 ± 7	12	25.6 ± 4.1	Current -5 Former − 9 Never − 6	NSPT +Local antibiotics	2 months
			Intervention group-2	21	48 ± 7	11	25.7 ± 3.5	Current − 6 Former − 11 Never − 4	NSPT	
			Control group	24	48 ± 6	15	25.3 ± 3.4	Current − 7 Former − 10 Never − 7	no treatment	
Tonetti 2007	UK	periodontitis	Intervention group	61	47.7 ± 7.9	30	27.2 ± 5.0	Current − 18 Former − 19 Never − 24	NSPT +Local antibiotics	6 months
			Control group	59	47.8 ± 6.3	30	27.3 ± 5.4	Current − 20 Former − 18 Never − 21	CPT	
Higashi 2009	Japan	periodontitis + CAD	Intervention group	24	64 ± 14	19	24.2 ± 3.2	Current − 0 Former − 0 Never − 24	NSPT +Systemic antibiotics	6 months
			Control group	24	63 ± 13	18	24.3 ± 3.2	Current − 0 Former − 0 Never − 24	no treatment	
Taylor 2010	Australia	periodontitis + Multip-morbidities	Intervention group	61	52.1 ± 13.3	29	NI	Current − 19	NSPT	3 months
			Control group	54	56.1 ± 12.1	28	NI	Current − 16	no treatment	
Li 2011	China	periodontitis	Intervention group	25	58.6 ± 11.6	10	24.3 ± 3.2	Never − 25	NSPT +Local antiseptic	3 months
			Control group	25	59.7 ± 10.3	13	23.2 ± 3.6	Never − 25	no treatment	
Kamil 2011	Jordan	periodontitis	Intervention group	18	46.7 ± 3.4	10	25.0 ± 1.6	NA	NSPT	3 months
			Control group	18	45.4 ± 3.3	10	24.8 ± 1.3	NA	no treatment	
Chen 2012	China	periodontitis + diabetes	Intervention group-1	42	59.86 ± 9.48	23	24.46 ± 2.82	NA	NSPT	6 months
			Intervention group-2	43	57.91 ± 11.35	26	23.88 ± 3.56	NA	NSPT	
			Control group	41	63.2 ± 8.51	17	23.51 ± 3.10	NA	no treatment	
Bokhari 2012	Pakistan	periodontitis + CHD	Intervention group	212	49.0 ± 0.6	180	28.3 ± 0.5	Current − 0 Former − 80 Never − 132	NSPT	2 months
			Control group	105	56.1 ± 12.1	93	28.4 ± 0.7	Current − 0 Former − 47 Never − 58	no treatment	
Wehmeyer 2013	America	periodontitis + Dialysis	Intervention group	25	54.1 ± 9.0	15	31.4 ± 8.3	Current − 3 Former − 7 Never − 15	NSPT	6 months
			Control group	26	52.7 ± 10.6	8	31.9 ± 6.4	Current − 4 Former − 8 Never − 14	no treatment	
Kapellas 2014	Australia	periodontitis + Diabetes (26.6%)	Intervention group	89	42.2 ± 10.5	96	29.06 ± 6.15	Current − 97 Former/Never- 53	NSPT	12 months
			Control group	79					no treatment	
Fang 2015	China	periodontitis ESRD	Intervention group	48	53.71 ± 5.89	28	20.56 ± 3.45	Current − 6 Former − 1 Never − 41	NSPT	6 months
			Control group	49	55.53 ± 6.7	27	20.94 ± 3.21	Current − 5 Former − 3 Never − 41	no treatment	
Fu 2016	China	perodontitis + hyperlipidemia	Intervention group	54	46.61 ± 10.07	26	24.16 ± 3.75	Current − 10 Former − 5 Never − 36	NSPT +Local antiseptic	6 months
			Control group	55	47.25 ± 8.94	29	23.25 ± 3.64	Current − 7 Former − 6 Never − 39	CPT	
Zhou 2017	China	periodontitis + prehypertention	Intervention group	53	41 ± 8.64	28	23.96 ± 3.87	NI	NSPT +Local antibiotic	6 months
			Control group	54	38.38 ± 9.31	28	23.55 ± 3.26	NI	CPT	
Saffi 2018	Brazil	periodontitis + CAD	Intervention group	31	58.6 ± 8.5	28	27 ± 3.6	Current-4 Former-20 Never-7	NSPT	3 months
			Control group	38	61.7 ± 8.3	24	28.2 ± 4.1	Current-4 Former-24 Never-10	CPT	
Montenegro 2019	Brazil	periodontitis + CAD	Intervention group	39	58.4 ± 9.2	8	27.5 ± 4.0	Current-3 Former-23 Never-13	NSPT	3 months
			Control group	43	60.8 ± 8.5	13	28.0 ± 4.0	Current-6 Former-25 Never-12	CPT	
Czesnikiewicz-Guzik 2019	Poland	periodontitis + hypertension	Intervention group	50	53(50–56)†	26	28(26.8–29.2)†	Current-17 Former-7 Never-26	NSPT +Local antibiotic	2 months
			Control group	51	55(54–58)†	31	29(28.1–30.7)†	Current-15 Former-10 Never-26	CPT	
Lobo 2020	Brazil	periodontitis + STEMI	Intervention group	24	52.7 ± 9.3	16	NI	smokers-19	NSPT	6 months
			Control group	24	54.6 ± 6.7	18	NI	smokers-22	no treatment	
Wang 2020	China	periodontitis + T2DM	Intervention group	29	64.4 ± 9.3	17	26.4 ± 3.0	smokers-3	NSPT	6 months
			Control group	29	63.7 ± 8.3	16	25.9 ± 3.5	smokers-4	no treatment	
Montero 2020	Spain	periodontitis + Met S	Intervention group	32	56.7 ± 6.5	22	39.1 ± 5.6	Current-8 Former-9 Never-15	NSPT +Systemic antibiotics	6 months
			Control group	31	58.3 ± 5.8	22	38.0 ± 4.7	Current-3 Former-17 Never-11	CPT	
Doke 2021	Japan	periodontitis + Met S	Intervention group	26	57.5 ± 10.0	NI	26.9 ± 3.3	NI	NSPT	3 months
			Control group	27	57.5 ± 10.0	NI	26.9 ± 3.6	NI	no treatment	

†Data are presented as mean(95% CI). CAD: Coronary artery disease; CHD: Coronary Heart Disease; CPT: Control Periodontal Treatment; ESRD: End Stage Renal Disease; Met S: Metabolic syndrome; NI: No Information; NSPT: Non-Surgical Periodontal Therapy; STEMI: ST-Segment Elevation Myocardial Infarction; T2DM: Type 2 Diabetes Mellitus

Details of the criteria used to define periodontitis and co-morbidity by each study are shown in Supplemental Table S3. Five studies recruited participants suffering only from periodontitis, while the rest included patients with co-morbidity. The co-morbidity can be categorized into three groups: CVD (ST-segment elevation myocardial infarction [STEMI], coronary artery disease [CAD], coronary heart disease [CHD], hypertension); metabolic disorder (diabetes, hyperlipidemia, metabolic syndrome [Met S]) or others (end-stage renal disease [ESRD]). All of the studies reported diagnosing a periodontal condition based on clinical examination. Eight studies included individuals with severe/advanced periodontitis, seven with moderate to severe/advanced periodontitis, five with mild, moderate to severe periodontitis, and one study with mild-moderate periodontitis.

Subjects in the intervention group received NSPT in the form of supragingival scaling, subgingival scaling and root surface debridement with or without adjunction antiseptic and/or antibiotics. The intervention protocols varied across studies regarding the number of NSPT sessions, duration of the clinical session, subgingival reintervention, or extraction of teeth with a poor prognosis and use of antiseptic /antibiotics (Supplemental Table S4).

### Risk of bias in included studies

**Fig. 2:**
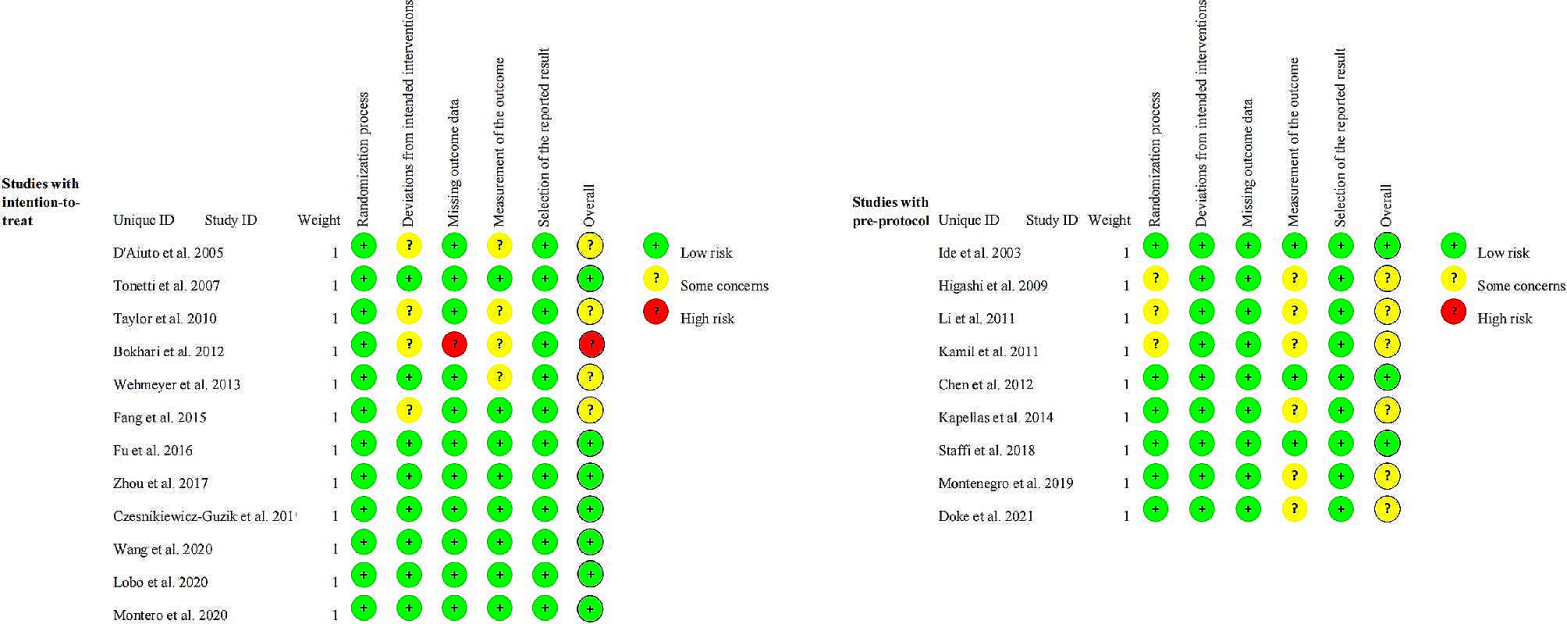
Summary of the risk of bias

Ten studies were categorized as low risk of bias, the other ten studies with some concerns, and one as high risk (Fig. 2). Most trials described their randomization process by using computer-generated random numbers and an opaque envelope method for allocation concealment. The main reasons for the risk of bias were the randomization process, the blinding of participants/personnel, and the loss of follow-up.

### Meta analyses

Effect of NSPT on the levels of systemic inflammation markers.

**Fig. 3:**
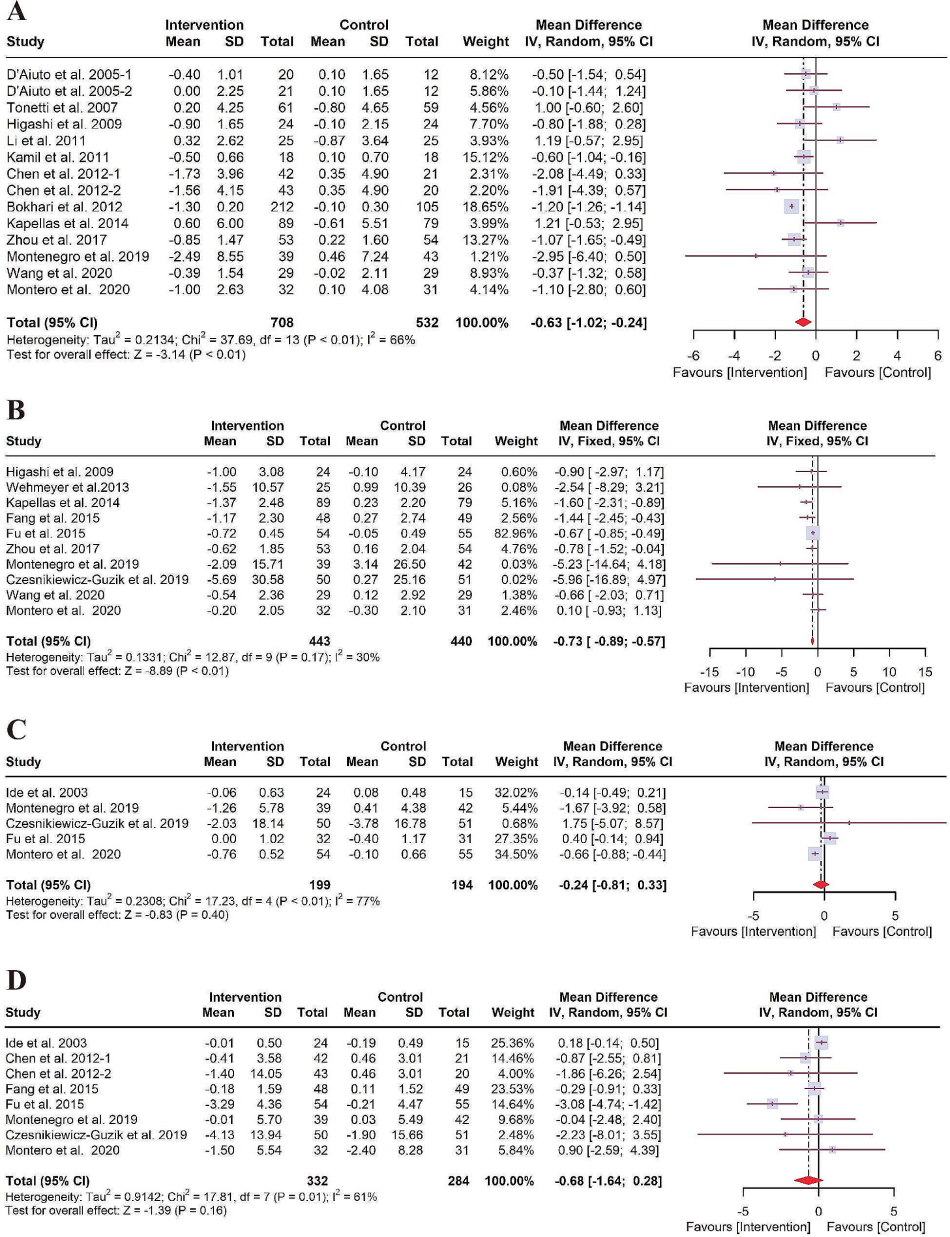
Forest plot for the levels of systemic inflammation markers. (**A**) CRP; (**B**) IL-6; (**C**) IL-1β; (**D**) TNF-α. CI: confidence interval; CRP: C-reactive protein; IL-6: Interleukin-6; IL-1β: Interleukin-1β; IV: Inverse variance; SD: standard deviation; TNF-α: tumor necrosis factor-alpha

Figure 3A shows a statistically significant reduction of CRP of -0.63 mg/L (95% CI: -1.02 to -0.24, *P* < 0.01) in the NSPT group compared with the control group. As for heterogeneity, the *I*^*2*^ was 66% (*P* < 0.01), representing moderate heterogeneity across included studies. Therefore, the result of the random effects model was presented. Figure 3B shows that NSPT is associated with a significant statistical reduction of IL-6 (WMD of -0.73 pg/mL, 95% CI: -0.89 to -0.57, *P* < 0.01, *I*^*2*^ = 30%, *P* = 0.17). Nevertheless, no significant difference in change of the levels of IL-1β (WMD of -0.24 pg/mL, 95% CI: -0.81 to 0.33, *P* = 0.40, *I*^*2*^ = 77%, *P* < 0.01) and TNF-α (WMD of -0.68 pg/mL, 95% CI: -1.64 to 0.28, *P* = 0.16, *I*^*2*^ = 61%, *P* = 0.01) were observed (Fig. 3C and D).

#### Effect of NSPT on the level of lipids

**Fig. 4:**
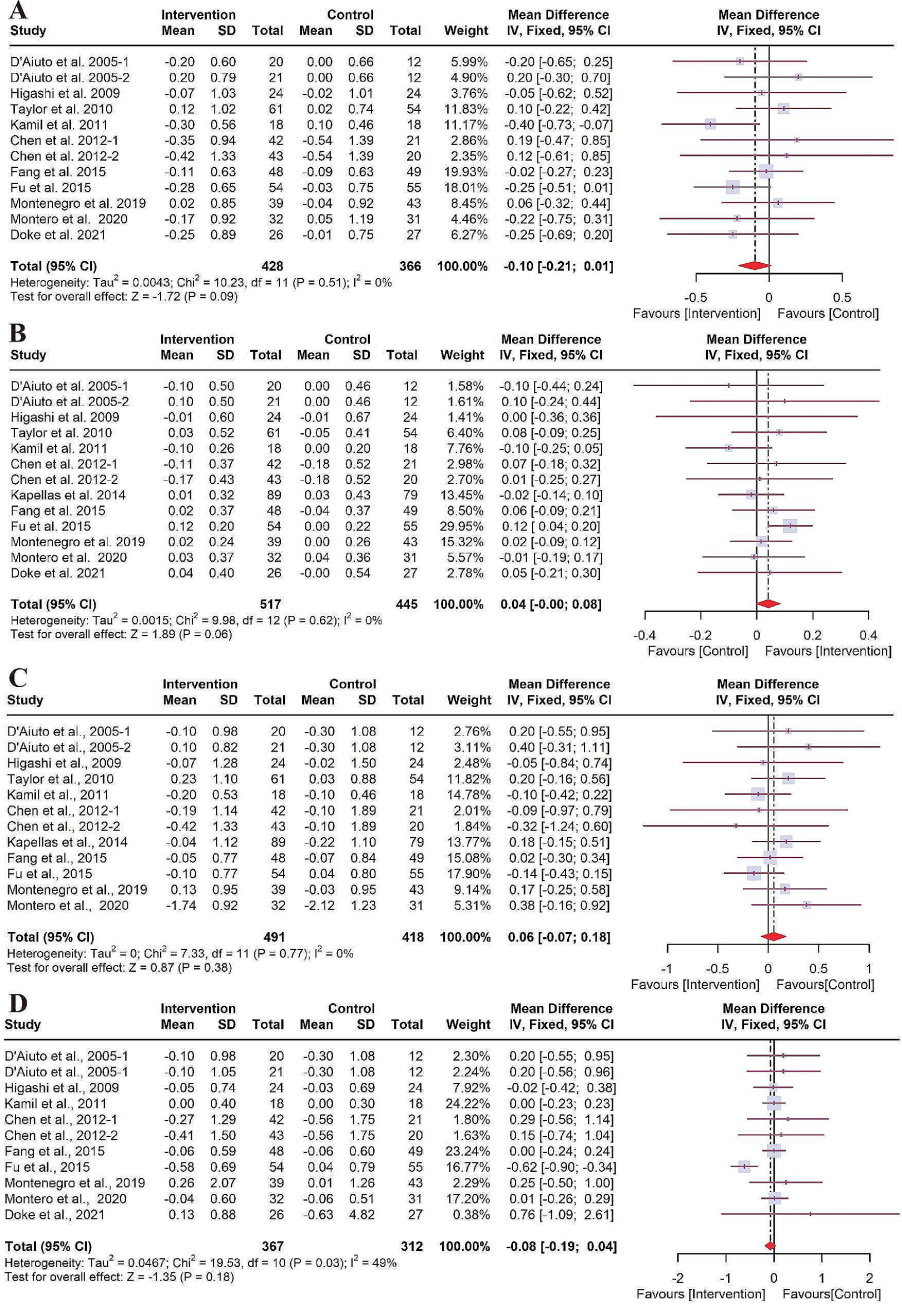
Forest plot for the levels of lipids. (**A**) LDL; (**B**) HDL; (**C**) TC; (**D**) TG. CI: confidence interval; HDL: high-density lipoprotein; IV: Inverse variance; LDL: low-density lipoprotein; SD: standard deviation; TC: total cholesterol; TG: triglycerides

As shown in Fig. 4, no significant difference in changes of lipid levels (LDL [WMD of -0.10 mM, 95% CI: -0.21 to 0.01, *P* = 0.09, *I*^*2*^ = 0%, *P* = 0.51], HDL [WMD of 0.04 mM, 95% CI: 0.00 to 0.08, *P* = 0.06, *I*^*2*^ = 0%, *P* = 0.62], TC[WMD of 0.06 mM, 95% CI: -0.07 to 0.18, *P* = 0.38, *I*^*2*^ = 0%, *P* = 0.77], TG[WMD of -0.08 mM, 95% CI: -0.19 to 0.04, *P* = 0.18, *I*^*2*^ = 49%, *P* = 0.03]) was found between intervention and control groups.

#### Effect of NSPT on the vascular function

**Fig. 5:**
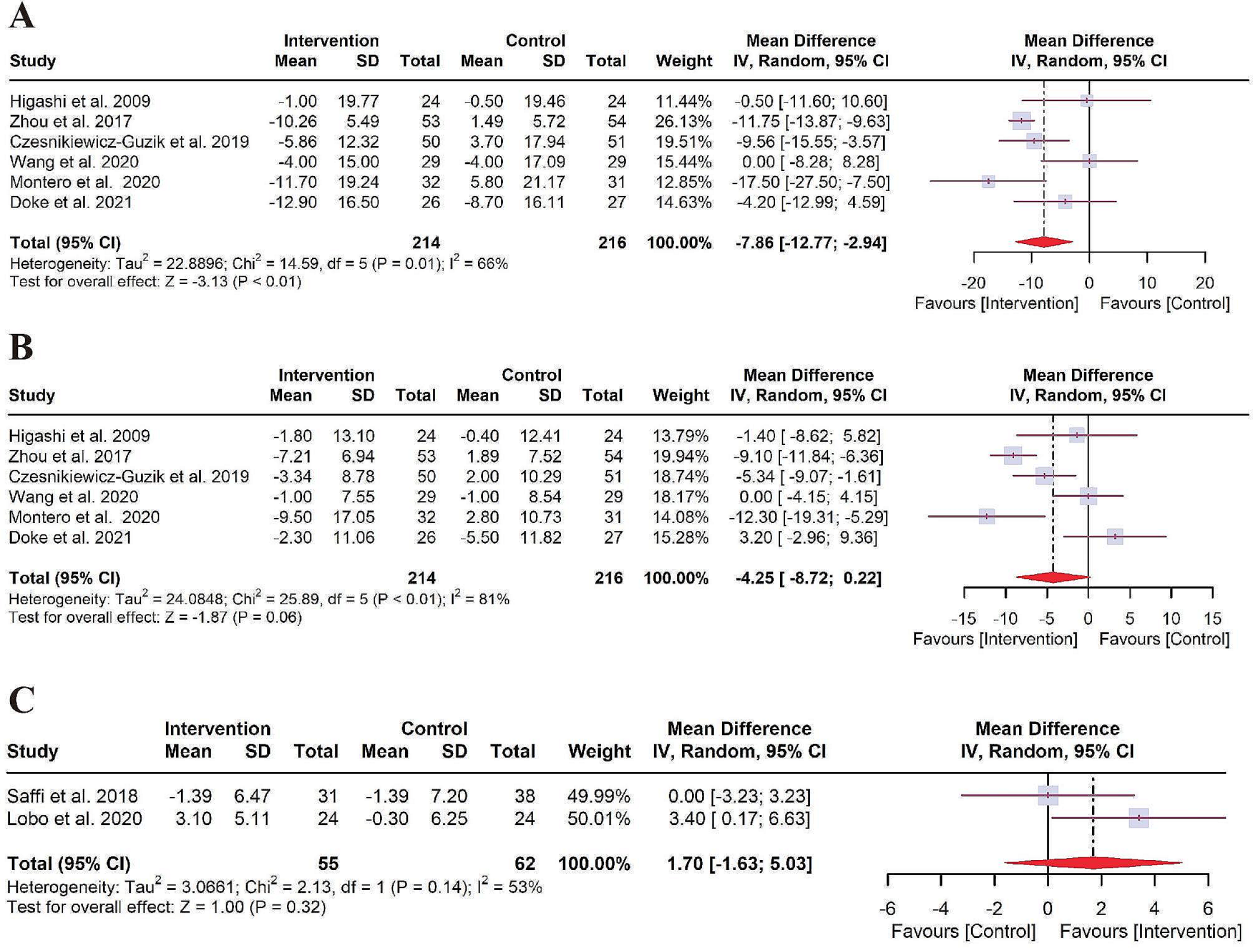
Forest plot for vascular function. (**A**) SBP; (**B**) DBP; (**C**) FMD. CI: confidence interval; DBP: diastolic blood pressure; FMD: flow-mediated dilatation; IV: Inverse variance; SBP: systolic blood pressure; SD: standard deviation

Six RCTs focused on SBP/DBP changes between the intervention and control groups. Owing to the presence of heterogeneity of SBP (*I*^*2*^ = 66%, *P* = 0.01) and DBP (*I*^*2*^ = 81%, *P* < 0.01), the random-effects model was adopted to calculate pooled WMD. The forest plot of SBP generated by random-effects model disclosed a pooled WMD of -7.85 mmHg (95% CI: -12.77 to -2.94, *P* < 0.01) (Fig. 5A). However, no significant difference in change of DBP was observed (WMD of -4.25 mmHg, 95% CI: -8.72 to 0.22, *P* = 0.06) (Fig. 5B). Additionally, the pooled estimate of the treatment effect on the FMD was 1.70% (95% CI: -1.63 to 5.03, *P* = 0.32, *I*^*2*^ = 53%, *P* = 0.14) (Fig. 5C), indicating that the increase in FMD after NSPT was not statistically significant compared to control.

#### Subgroup analyses

Subgroup analyses were conducted according systemic health status, use of antibiotics, and the time of follow-up. Regarding the impact of systemic health status, Supplemental Table S5 showed a statistically significant subgroup effect of CRP (*P* = 0.02). The subgroup analysis of CRP showed a statistically significant reduction of 1.20 mg/L for NSPT in CVD patients. A similar trend was noted in studies including participants with metabolic disorders. However, no statistically significant reduction of CRP was observed in periodontitis patients without any comorbidities (otherwise healthy). As for the usage of antiseptic and/or antibiotics, significant differences between groups were found in IL-6 (*P* = 0.01), TG (*P* = 0.03), SBP (*P* < 0.01), and DBP (*P* < 0.01) as well (Supplemental Table S6). The results of the subgroup that used antiseptic and/or antibiotics showed statistically significant reductions in TG, SBP and DBP. Conversely, a strong increase of IL-6 level was noted in subgroup with antiseptic and/or antibiotics use (Supplemental Table S6). When grouping studies according to the duration of follow-up (< 6 months vs. ≥ 6 months), there is no statistically significant intervention effect between groups (Supplemental Table S7).

#### Sensitivity analyses

As presented in Supplemental Fig. S1-S3, when a study was excluded, the pooled estimates did not change substantially, indicating that no single study significantly impacted on the pooled estimate. Further sensitivity analyses by excluding the studies in which the control group performed CPT suggested that the pooled estimate of the outcomes was robust and not influenced excessively (Supplemental Fig. S4-S6).

#### Publication bias

There was no publication bias of IL-6, LDL, HDL, TC, and TG in the statistical or visual appraisal of funnel plots (Supplemental Fig. S5B-F). The funnel plot and Egger’s test (*P* = 0.02 of CRP were asymmetric (Supplemental Fig. S5A), suggesting that these studies included are subject to publication bias.

#### Certainty in the body of evidence

Treatment of periodontitis effected IL-6, LDL, HDL, TC, TG and SBP with moderate certainty of evidence while the certainty of evidence on CRP, IL-1β, TNF-α, DBP and FMD was low (Appendix S3).

## Discussion

### Principal findings

In this systemic review and meta-analysis, we found that NSPT decreased CRP, IL-6, and SBP of patients with periodontitis, while there was no difference in outcomes, including IL-1β, TNF-α, LDL, HDL, TC, TG, DBP, and FMD. In subgroup analyses, the effect of NSPT on different cardiovascular disease risk markers differed by systemic health status, and antiseptic/antibiotics use. Patients with periodontitis, CVD, or metabolic disorder benefited more from NSPT, and adjuvant use of antiseptic/antibiotics can enhance the effect of treatment.

### Comparison with other studies

The findings from this review are the most recent and comprehensive assessment of the available data from only RCTs to report the effect of NSPT on CVD risk markers in patients with periodontitis. In contrast, the majority of previously relevant meta analyses only included periodontitis patients with specific systemic health conditions, such as CVD [75], CAD [76], hypertension [77] and diabetes [78].

In this study, NSPT significantly decreased CRP levels in patients with periodontitis. Notably, subgroup analysis based on the health status of patients found that compared with the control group, there was a statistically significant difference in periodontitis patients with CVD, suggesting that periodontitis patients with CVD benefited most from NSPT. A recent meta-analysis that focused on CRP values alone concluded that treatment of periodontitis reduces serum CRP levels (up to 6 months follow-up), and no treatment effect was observed at 12 months or beyond [79]. Similarly, this study found a statistically significant reduction in CRP levels after < 6 months follow-up, while, with ≥ 6 months follow-up, the differences were not statistically significant. Among the interleukins released during inflammatory processes, IL-6, IL-8, and IL-1β have been widely explored in periodontal medicine for their possible joint pathogenic involvement in periodontitis and other systemic inflammatory conditions. There have been conflicting results in the past about how periodontal therapy affects IL-6 levels in patients with periodontitis. Some claimed a significant reduction [16, 75], while others reported a similar lack of effect to our study [14]. In this study, IL-6 levels were significantly lower in patients with periodontitis after NSPT compared to the control group. In addition, this study found that there was no significant improvement in IL-1β and TNF-α in periodontitis patients after NSPT, which may be related to the small sample size.

In line with our findings, a meta-analysis published in 2022 confirmed a statistically significant reduction in SBP after NSPT vs. CPT among periodontitis patients [22]. The difference is that in this study, no significant reduction in DBP was found in periodontitis patients after NSPT. The reason may be that they only included patients with periodontitis combined with hypertension/prehypertension. CVD is inseparable from endothelial inflammation, and severe endothelial dysfunction is a significant factor affecting adverse cardiovascular events [80]. Our study revealed a non-statistically significant rise in FMD as a result of NSPT. It has been proposed that NSPT has a positive tendency to stop future deterioration by preventing the formation of inflammation, even though it does not appear to have any therapeutic effect on vascular dysfunction. This is because proinflammatory stimuli significantly influence endothelial cell damage and apoptosis [81]. Contrary to the findings of this investigation, endothelial function indicators, such as FMD and forearm blood flow (FBF), have been found to be considerably improved by periodontal treatment in periodontitis patients with CVD [82].

A previous meta-analysis [75, 77] found that, NSPT would appear unrelated to improvements in LDL, HDL-C, TC, and TG levels, consistent with our findings. Interestingly, in subgroup analyses, the periodontitis patients with metabolic disorders treated with NSPT showed significant improvement in TG and HDL compared to controls. This is most likely explained by higher baseline TG levels or lower baseline HDL levels for the periodontitis patient groups with metabolic disorders versus those without metabolic disorders. If these parameters can be improved by periodontal therapy, this could constitute a beneficial strategy for preventing CVD, as these are some of the main components of atheromatous plaque [19].

### Potential mechanisms

Periodontitis is a chronic inflammatory disease caused by dysbiosis between the host and the oral bacterial communities [82]. Periodontal bacterial lipopolysaccharides stimulate monocytes to generate inflammatory mediators such as TNF-α, interleukins, and proteolytic enzymes like matrix metalloproteinases. In addition to the periodontal lesion, this inflammatory response to the bacterial assault also impacts other parts of the body [19]. The migration of periodontal bacteria into the circulatory system (bacteremia) and the higher levels of systemic inflammation caused by periodontitis have been proposed as the mechanisms behind the link between periodontitis and CVDs [18, 19, 83]. Frequent episodes of bacteremia are experienced by subjects with periodontitis, especially after dental prophylaxis, scaling, extraction of teeth, surgical extraction of third molars, and periodontal probing, in addition to daily life activities like brushing, flossing, and biting [83]. In atherothrombotic tissues, periodontal inflammation and viable bacteria have been found [18, 83, 84]. As proven in experimental pre-clinical studies, these bacteria and their products and virulence factors may influence the pathogenesis of atherosclerosis [17].

### Strengths, limitations and recommendations

The major strength of this study is that we comprehensively and systematically studied the effect of NSPT on markers related to CVD in patients suffering from periodontitis to obtain a deeper understanding of the effect of periodontal therapy on systemic health. Furthermore, three subgroups were established to explore the effects of different systemic health status, different interventions (with or without usage of adjunctive antiseptic and/or antibiotics), and different follow-up time on outcomes, to explore the effects of NSPT on risk markers of CVD from multiple perspectives. Finally, there were a sizable number of participants from five continents in this study. This may make our findings more broadly extrapolated.

A few limitations warrant mentioning. First, the heterogeneity of most outcomes was moderate to large heterogeneity. Potential sources of heterogeneity include varying diagnostic criteria for periodontitis, inclusion of periodontitis patients of various severity, different smoking status and different intervention procedures between studies. Second, the relationship between CVD and periodontitis involves multiple confounding factors, such as BMI, smoking, and gender, which may have influenced the study results. Unfortunately, we tried to analyze by controlling factors such as BMI, smoking, and gender, but failed to conduct further analysis due to the inability to unify standards. Additionally, although this meta-analysis included rigorous inclusion/exclusion criteria and an analysis of publication bias, but the results must be interpreted carefully. This meta-analysis for IL-1β, TNF-α, SBP, DBP and FMD included limited number of studies. This factor may have caused discrepancies, and the results of this meta-analysis should be accepted with caution.

There is a growing body of studies examining the association between periodontitis and CVD. Due to the great significance of answering clinical questions related to the effect of periodontal treatment on CVD, it is important to highlight the limitations and the gaps of the existing literature to improve the design and the validity of future studies. Future studies should include a detailed description of the recruitment process and sample selection, and present effects adjusted at least for age, sex and smoking status, which may contribute to between-study heterogeneity. Given the variations in the intervention procedures used by each study, it is essential to emphasize the severity of periodontitis as determined by case criteria, the effectiveness of periodontal therapy in clearing the periodontal inflammation, and the role this inflammation plays in the systemic inflammation burden. Another important issue that should be raised is the need for adequate and transparent reporting of methodology and findings in future studies to improve the applicability of the evidence. As needed, more RCTs with longer-term monitoring and follow-up are advised. In a similar vein, certain confounding variables like smoking and co-morbidities need to be closely managed.

## Conclusion

In conclusion, moderate certainty evidence shows that NSPT has a positive effect on the reduction of IL-6 and SBP in patients with periodontitis, while low certainty evidence shows that NSPT is effective for reduction of CRP. Moderate certainty evidence showed that NSPT did not show a positive effect on LDL, HDL, TG and TC, and low certainty evidence showed that NSPT did not show a positive effect on IL-1β, TNF-α, DBP, and FMD.

### Electronic supplementary material

Below is the link to the electronic supplementary material.

Supplementary Material 1

Supplementary Material 2

## Data Availability

The data supporting this study’s findings are available from the corresponding author upon reasonable request.
