# Correction: Effect of non-surgical periodontal therapy on risk markers of cardiovascular disease: a systematic review and meta-analysis

**DOI:** 10.1186/s12903-024-04559-1

**Published:** 2024-07-18

**Authors:** Rijing Meng, Jialei Xu, Chenrui Fan, Haiqing Liao, Zeni Wu, Qixin Zeng

**Affiliations:** 1 Department of Periodontics and Oral Medicine, College & Hospital of Stomatology, Guangxi Medical University, No. 22, Shuangyong Road, Qingxiu District, Guangxi Nanning, 530021 China; 2 Guangxi Key Laboratory of Oral and Maxillofacial Rehabilitation and Reconstruction, Nanning, China; 3 Guangxi Health Commission Key laboratory of prevention and treatment for oral infectious diseases, Nanning, China; 4 School of Population Medicine and Public Health, Chinese Academy of Medical Sciences, Peking Union Medical College, Beijing, 100730 China

**Correction to: BMC Oral Health (2024) 24:692**

10.1186/s12903-024-04433-0

In this article [1], the funding number was incorrectly stated as the Nanning Qingxiu district science and technology plan project (Grant No.KY017005) instead of the correct funding number, which is the Nanning Qingxiu district science and technology plan project (Grant No. 2016059).
