# Supplementary material for: Effect of non-surgical periodontal therapy on risk markers of cardiovascular disease: a systematic review and meta-analysis
Source: BMC Oral Health. 2024 Jun 14;24:692. doi: 10.1186/s12903-024-04433-0 (PMC11177403; doi:10.1186/s12903-024-04433-0)
Supplement: Supplementary file 1 — Supplementary Material 1 [file 12903_2024_4433_MOESM1_ESM.docx]

**List of Supplemental Table**

**Supplemental Table S1: Search strategy - All databases**

**Supplemental Table S2: List of excluded studies**

**Supplemental Table S3: Diagnosis of periodontitis and comorbidity**

**Supplemental Table S4: The intervention protocols of included studies**

**Supplemental Table S5: Subgroup analysis according to systemic health status.**

**Supplemental Table S6: Subgroup analysis according to usage of antiseptic/antibiotics.**

**Supplemental Table S7: Subgroup analysis according to follow-up time.**

**Supplemental Table S1: Search strategy - All databases**

**Database: PubMed**

| ID | Search |
| --- | --- |
| #1 | "Periodontitis"[MeSH Terms] |
| #2 | "Periodontitides"[Title/Abstract] |
| #3 | "Pericementitis"[Title/Abstract] |
| #4 | "Periodontal Diseases"[MeSH Terms] |
| #5 | "disease periodontal"[Title/Abstract] |
| #6 | "diseases periodontal"[Title/Abstract] |
| #7 | "periodontal disease"[Title/Abstract] |
| #8 | "Parodontosis"[Title/Abstract] |
| #9 | "Parodontoses"[Title/Abstract] |
| #10 | "pyorrhea alveolaris"[Title/Abstract] |
| #11 | "Periodontitis"[MeSH Terms] OR "Periodontitides"[Title/Abstract] OR "Pericementitis"[Title/Abstract] |
| #12 | "Periodontal Diseases"[MeSH Terms] OR "disease periodontal"[Title/Abstract] OR "diseases periodontal"[Title/Abstract] OR "periodontal disease"[Title/Abstract] OR "Parodontosis"[Title/Abstract] OR "Parodontoses"[Title/Abstract] OR "pyorrhea alveolaris"[Title/Abstract] |
| #13 | "periodontitis/therapy"[MeSH Terms] |
| #14 | "periodontal diseases/therapy"[MeSH Terms] |
| #15 | "periodontal therapy"[Title/Abstract] |
| #16 | "periodontal treatment"[Title/Abstract] |
| #19 | "periodontal intervention"[Title/Abstract] |
| #20 | "periodontitis/therapy"[MeSH Terms] OR "periodontal diseases/therapy"[MeSH Terms] OR "periodontal therapy"[Title/Abstract] OR "periodontal treatment"[Title/Abstract] OR "periodontal intervention"[Title/Abstract] |
| #21 | "Periodontitis"[MeSH Terms] OR "Periodontitides"[Title/Abstract] OR "Pericementitis"[Title/Abstract] OR "Periodontal Diseases"[MeSH Terms] OR "disease periodontal"[Title/Abstract] OR "diseases periodontal"[Title/Abstract] OR "periodontal disease"[Title/Abstract] OR "Parodontosis"[Title/Abstract] OR "Parodontoses"[Title/Abstract] OR "pyorrhea alveolaris"[Title/Abstract] OR "periodontitis/therapy"[MeSH Terms] OR "periodontal diseases/therapy"[MeSH Terms] OR "periodontal therapy"[Title/Abstract] OR "periodontal treatment"[Title/Abstract] OR "periodontal intervention"[Title/Abstract] |
| #22 | "Cardiovascular Diseases"[MeSH Terms] |
| #23 | "cardiovascular disease"[Title/Abstract] |
| #24 | "disease cardiovascular"[Title/Abstract] |
| #25 | "diseases cardiovascular"[Title/Abstract] |
| #26 | "Cardiovascular Diseases"[MeSH Terms] OR "cardiovascular disease"[Title/Abstract] OR "disease cardiovascular"[Title/Abstract] OR "diseases cardiovascular"[Title/Abstract] |
| #27 | "Atherosclerosis"[MeSH Terms] |
| #28 | "Atheroscleroses"[Title/Abstract] |
| #29 | "Atherogenesis"[Title/Abstract] |
| #30 | "Atherosclerosis"[MeSH Terms] OR "Atheroscleroses"[Title/Abstract] OR "Atherogenesis"[Title/Abstract] |
| #31 | "Cerebrovascular Disorders"[MeSH Terms] |
| #32 | "Cardiovascular Diseases"[MeSH Terms] OR "cardiovascular disease"[Title/Abstract] OR "disease cardiovascular"[Title/Abstract] OR "diseases cardiovascular"[Title/Abstract] OR "Atherosclerosis"[MeSH Terms] OR "Atheroscleroses"[Title/Abstract] OR "Atherogenesis"[Title/Abstract] OR "Cerebrovascular Disorders"[MeSH Terms] |
| #33 | "randomized controlled trial"[Publication Type] OR "randomized"[Title/Abstract] OR "placebo"[Title/Abstract] |
| #34 | ("Periodontitis"[MeSH Terms] OR "Periodontitides"[Title/Abstract] OR "Pericementitis"[Title/Abstract] OR ("Periodontal Diseases"[MeSH Terms] OR "disease periodontal"[Title/Abstract] OR "diseases periodontal"[Title/Abstract] OR "periodontal disease"[Title/Abstract] OR "Parodontosis"[Title/Abstract] OR "Parodontoses"[Title/Abstract] OR "pyorrhea alveolaris"[Title/Abstract]) OR ("periodontitis/therapy"[MeSH Terms] OR "periodontal diseases/therapy"[MeSH Terms] OR "periodontal therapy"[Title/Abstract] OR "periodontal treatment"[Title/Abstract] OR "periodontal intervention"[Title/Abstract])) AND ("Cardiovascular Diseases"[MeSH Terms] OR "cardiovascular disease"[Title/Abstract] OR "disease cardiovascular"[Title/Abstract] OR "diseases cardiovascular"[Title/Abstract] OR ("Atherosclerosis"[MeSH Terms] OR "Atheroscleroses"[Title/Abstract] OR "Atherogenesis"[Title/Abstract]) OR "Cerebrovascular Disorders"[MeSH Terms]) AND ("randomized controlled trial"[Publication Type] OR "randomized"[Title/Abstract] OR "placebo"[Title/Abstract]) |

**Database: the Cochrane library**

| ID | Search |
| --- | --- |
| #1 | MeSH descriptor: [Periodontitis] explode all trees |
| #2 | (Periodontitides):ti,ab,kw OR (Pericementitis):ti,ab,kw (Word variations have been searched) |
| #3 | MeSH descriptor: [Periodontal Diseases] explode all trees |
| #4 | (Disease, Periodontal):ti,ab,kw OR (Diseases, Periodontal):ti,ab,kw OR (Periodontal Disease):ti,ab,kw OR (Parodontosis):ti,ab,kw OR (Parodontoses):ti,ab,kw (Word variations have been searched) |
| #5 | (Pyorrhea Alveolaris):ti,ab,kw (Word variations have been searched) |
| #6 | (Periodontal therapy):ti,ab,kw OR (Periodontal treatment):ti,ab,kw OR (Periodontal intervention):ti,ab,kw (Word variations have been searched) |
| #7 | #1 OR #2 OR #3 OR #4 OR #5 OR #6 |
| #8 | MeSH descriptor: [Cardiovascular Diseases] explode all trees |
| #9 | (Cardiovascular Disease):ti,ab,kw OR (Disease, Cardiovascular):ti,ab,kw OR (Diseases, Cardiovascular):ti,ab,kw (Word variations have been searched) |
| #10 | #8 OR #9 |
| #11 | MeSH descriptor: [Atherosclerosis] explode all trees |
| #12 | (Atheroscleroses):ti,ab,kw OR (Atherogenesis):ti,ab,kw (Word variations have been searched) |
| #13 | #11 OR #12 |
| #14 | MeSH descriptor: [Cerebrovascular Disorders] explode all trees |
| #15 | #10 OR #13 OR #14 |
| #16 | #7 AND #15 |

**Database: Web of Science**

| ID | Search |
| --- | --- |
| #1 | TS=(Periodontitis) |
| #2 | (TS=(Periodontitides)) OR TS=(Pericementitis) |
| #3 | #1 OR #2 |
| #4 | ((((TS=(Periodontal disease*)) OR TS=(Disease*, Periodontal)) OR TS=(Parodontosis)) OR TS=(Parodontoses )) OR TS=(Pyorrhea Alveolaris) |
| #5 | ((TS=(Periodontal therapy)) OR TS=(Periodontal treatment)) OR TS=(Periodontal intervention) |
| #6 | #3 OR #4 OR #5 |
| #7 | TS=(Cardiovascular Diseases) |
| #8 | (TS=(Cardiovascular Disease)) OR TS=(Disease*, Cardiovascular) |
| #9 | #7 OR #8 |
| #10 | ((TS=(Atherosclerosis)) OR TS=(Atheroscleroses)) OR TS=(Atherogenesis) |
| #11 | (TS=(cerebrovascular Diseases)) OR TS=(Cerebrovascular Disorders) |
| #12 | #9 OR #10 OR #11 |
| #13 | ((TS=(random* controlled trial)) OR TS=(random*)) OR TS=(placebo) |
| #14 | #6 AND #12 AND #13 |

**Database: Embase**

| #1 | 'periodontitis'/exp |
| --- | --- |
| #2 | 'periodontitides':ab,ti |
| #3 | 'pericementitis':ab,ti |
| #4 | 'periodontal disease'/exp |
| #5 | 'disease, periodontal':ab,ti |
| #6 | 'diseases, periodontal':ab,ti |
| #7 | 'periodontal disease':ab,ti |
| #8 | 'parodontosis':ab,ti |
| #9 | 'parodontosis':ab,ti |
| #10 | 'pyorrhea alveolaris':ab,ti |
| #11 | #1 OR #2 OR #3 |
| #12 | #4 OR #5 OR #6 OR #7 OR #8 OR #9 OR #10 |
| #13 | 'periodontal treatment':ab,ti |
| #14 | 'periodontal therapy':ab,ti |
| #15 | 'periodontal intervention':ab,ti |
| #16 | #13 OR #14 OR #15 |
| #17 | #11 OR #12 OR #16 |
| #18 | 'cardiovascular disease'/exp |
| #19 | 'cardiovascular disease':ab,ti |
| #20 | 'disease, cardiovascular':ab,ti |
| #21 | 'diseases, cardiovascular':ab,ti |
| #22 | 'atherosclerosis'/exp |
| #23 | 'atheroscleroses':ab,ti |
| #24 | 'atherogenesis':ab,ti |
| #25 | 'cerebrovascular disease'/exp |
| #26 | #18 OR #19 OR #20 OR #21 |
| #27 | #22 OR #23 OR #24 |
| #28 | #25 OR #26 OR #27 |
| #29 | 'random':ab,ti OR 'placebo':ab,ti OR 'double-blind':ab,ti |
| #30 | #17 AND #28 AND #29 |

**Supplemental Table S2: List of excluded studies**

| **Study** | **Title** | **Reasons for exclusion** |
| --- | --- | --- |
| D'Aiuto et al. 2006 | Periodontal infections cause changes in traditional and novel cardiovascular risk factors: Results from a randomized controlled clinical trial | Individuals were randomized either to a standard course of periodontal therapy (subgingival scaling and root planing) or an intensive one (including the adjunctive use of a locally delivered antimicrobial). Not matching criteria. |
| Lösche et al. 2007 | Periodontitis and Cardiovascular Disease: Periodontal Treatment Lowers Plasma Cholesterol | A review article, not RCTs. |
| Oz et al. 2007 | Beneficial Effects of Periodontal Treatment on Metabolic Control of Hypercholesterolemia | Periodontal treatment included periodontal surgery. |
| Vidal et al. 2009 | Periodontal Therapy Reduces Plasma Levels of Interleukin-6, C-Reactive Protein, and Fibrinogen in Patients with Severe Periodontitis and Refractory Arterial Hypertension | Not RCTs. |
| Offenbacher et al. 2009 | Results From the Periodontitis and Vascular Events (PAVE) Study: A Pilot Multicentered, Randomized, Controlled Trial to Study Effects of Periodontal Therapy in a Secondary Prevention Model of Cardiovascular Disease | Detailed data not available. |
| Sun et al. 2010 | Changes of adiponectin and inflammatory cytokines after periodontal intervention in type 2 diabetes patients with periodontitis | Periodontal treatment included periodontal surgery. |
| Skilton et al. 2011 | The effect of a periodontal intervention on cardiovascular risk markers in Indigenous Australians with periodontal disease: the PerioCardio study | A study protocol, not RCTs |
| Gunupati et al. 2011 | Effect of Phase I Periodontal Therapy on Anti-Cardiolipin Antibodies in Patients With Acute Myocardial Infarction Associated With Chronic Periodontitis | A cross-sectional study, not RCTs |
| López et al. 2012 | Effects of Periodontal Therapy on Systemic Markers of Inflammation in Patients With Metabolic Syndrome: A Controlled Clinical Trial | Data in graphs no value given. |
| Koppolu et al. 2013 | Estimate of CRP and TNF-alpha level before and after periodontal therapy in cardiovascular disease patients | Not RCTs |
| Caúla et al. 2014 | The effect of periodontal therapy on cardiovascular risk markers: a 6-month randomized clinical trial | Results were reported as Median (first quartile; third quartile). |
| Javed et al. 2014 | Effect of nonsurgical periodontal therapy (with or without oral doxycycline delivery) on glycemic status and clinical periodontal parameters in patients with prediabetes: a short-term longitudinal randomized case–control study | Patients were randomly divided into two groups: in group 1, scaling and root planing was performed, and in group 2, patients underwent scaling and root planing and oral doxycycline (100 mg) administration once daily for 15 days. Not matching criteria |
| Chou et al. 2015 | Major Adverse Cardiovascular Events in Treated Periodontitis: A Population-Based Follow-Up Study from Taiwan | A retrospective cohort study, not an RCTs. |
| Tawfig et al. 2015 | Effects of non‑surgical periodontal therapy on serum lipids and C‑reactive protein among hyperlipidemic patients with chronic periodontitis | Not RCTs |
| Hada et al. 2015 | Effect of Non-Surgical Periodontal Treatment on Clinical and Biochemical Risk Markers of Cardiovascular Disease: A Randomized Trial | Participants included gingivitis patients, Not matching criteria. |
| Jamieson et al. 2015 | Periodontal disease and chronic kidney disease among Aboriginal adults; an RCT | A study protocol, not RCTs. |
| Masi et al. 2018 | Mitochondrial oxidative stress, endothelial function and metabolic control in patients with type II diabetes and periodontitis: A randomised controlled clinical trial | Periodontal treatment included periodontal surgery. |
| D'Aiuto et al. 2018 | Systemic effects of periodontitis treatment in patients with type 2 diabetes: a 12 month, single-centre, investigator-masked, randomised trial | Periodontal treatment included periodontal surgery. |
| Kocher et al. 2019 | Effect of Periodontal Treatment on HbA1c among Patients with Prediabetes | No outcome of interest, not matching criteria. |
| Seinost et al. 2020 | Periodontal treatment and vascular inflammation in patients with advanced peripheral arterial disease: A randomized controlled trial | Data in graphs no value given. |
| Rapone et al. 2022 | The Impact of Periodontal Inflammation on Endothelial Function Assessed by Circulating Levels of Asymmetric Dimethylarginine: A Single-Blinded Randomized Clinical Trial | Periodontal treatment included periodontal surgery. |

RCTs：randomized clinical trials.

**Supplemental Table S3: Diagnosis of periodontitis and comorbidity**

| **Study** | **Periodontitis Classification** | **Periodontitis Diagnostic Criteria** | **Minimum Number of Teeth** | **Co-morbidity Diagnostic Criteria** |
| --- | --- | --- | --- | --- |
| Ide et al. 2003 | moderate to advanced chronic periodontitis | ≥ 5 teeth with PD ≥ 5mm, radiographic evidence of alveolar bone loss | ≥ 20 | With otherwise healthy |
| D'Aiuto et al. 2005 | generalized severe chronic periodontitis | PPD sites > 6mm, marginal alveolar bone loss > 30% and ≥ 50% teeth affected | NI | With otherwise healthy |
| Tonetti et al. 2007 | generalized severe periodontitis | PPD sites > 6mm, marginal alveolar bone loss > 30% and ≥ 50% teeth affected | NI | With otherwise healthy |
| Higashi et al. 2009 | Chronic periodontitis | ≥ 2 teeth with PPD ≥ 4 mm and CAL ≥ 3 mm | NI | **coronary artery disease (CAD)**  stenosis ≥ 70% in at least one proximal epicardial coronary artery and with objective evidence of myocardial infarction or at least one coronary stenosis ≥ 80% and classic angina without provocative testing |
| Taylor et al. 2010 | Periodontitis | ≥ sites with ≥ 5 mm PPD and ≥ 2 mm CAL | NI | History of hyperlipidemia  History of diabetes mellitus  History of hypertension |
| Li et al. 2011 | moderate-to-severe chronic periodontitis | fulfilled any of the following criteria：  > 6 sites with PD ≥ 4 mm;  > 25% of sites with interproximal CAL ≥ 5 mm;  ≥ 8 missing teeth due to chronic periodontitis excluding the third molars. | NI | With otherwise healthy |
| Kamil et al. 2011 | Advanced periodontitis | ≥ 6 teeth with PPD > 5mm and CAL ≥ 3 mm in three sites of each involved tooth | ≥ 20 | With otherwise healthy |
| Chen et al. 2012 | Chronic periodontitis  (Slight, moderated, and severe) | ≥ 1 mm mean CAL | ≥ 16 | **Type 2 diabetic** |
| Bokhari et al. 2012 | Periodontitis | ≥ 4 teeth with ≥ 1 sites with PPD ≥ 4mm and CAL ≥ 3 mm at the same site | ≥ 14 | **Coronary heart disease (CHD)**  > 50% stenosis of ≥ 1 coronary artery documented by coronary angiography |
| Wehmeyer et al. 2013 | Moderate/severe periodontitis | ≥ 2 teeth with 6mm CAL and ≥ one site with PD ≥ 5 mm | ≥ 16 | **Dialysis patients** |
| Kapellas et al. 2014 | Moderate Periodontitis | ≥2 interproximal sites with CAL ≥4 mm or ≥2 interproximal sites with PD ≥5 mm | NI | **Diabetes mellitus** 35%-27% |
| Fang et al. 2015 | Chronic periodontitis  (Slight, moderated, and severe) | ≥ 1 mm mean CAL | ≥16 | **End-stage renal disease (ESRD)**  Undergoing on average 4 h of haemodialysis 3 times/week. |
| Fu et al. 2016 | Moderate-to-advanced chronic periodontitis | 4 teeth with PPD > 4 mm, CAL ≥ 2 mm and radiographic evidence of bone loss. | ≥ 20 | **Hyperlipidemia**  meet at least one of the following criteria: TG > 2.26 mmol/L; HDL < 1.04 mmol/L;  or LDL > 4.14 mmol/L |
| Zhou et al. 2017 | Moderate to severe periodontitis | moderate periodontitis:  two sites between adjacent teeth with ≥ 4 mm CAL or ≥ 2 such sites with ≥ 5 mm PPD,  severe periodontitis  ≥ 2 sites between adjacent teeth with ≥6 mm CAL and ≥ one PPD ≥5 mm | NI | **Prehypertension** |
| Staffi et al. 2018 | Severe chronic periodontitis | ≥ two non-adjacent teeth with PD ≥5 mm and CAL ≥ 6 mm. | ≥ 10 | **Coronary artery disease (CAD)**  a clinical history of at least one documented of the following episodes:  acute coronary syndrome ≥ 6 months prior to trial inclusion; percutaneous/surgical revascularization; coronary angiography showing ≥ 50% lesion in at least one artery; or positive noninvasive testing of ischemia |
| Montenegro et al. 2019 | Severe periodontitis | ≥ 2 teeth with PD ≥ 5mm and CAL ≥ 6mm. | ≥10 | **Coronary artery disease (CAD)**  Occurrence of one or more of following events 6 months before entering the study:  History of myocardial infarction, stable angina or ischaemia in noninvasive tests; surgical or percutaneous myocardial revascularization and lesion size of greater than 50% in at least one major coronary artery, as assessed by angiography; presence of angina and positive results of noninvasive testing of ischaemia. |
| Czesnikiewicz-Guzik et al. 2019 | Moderate to severe periodontitis | moderate periodontitis:  two sites between adjacent teeth with ≥ 4 mm CAL or ≥ 2 such sites with ≥ 5 mm PPD,  severe periodontitis  ≥ 2 sites between adjacent teeth with ≥ 6 mm CAL and ≥ one PPD ≥ 5 mm | NI | **Hypertension** |
| Lobo et al. 2020 | Severe periodontitis | ≥ 5 teeth with ≥ 4mm CAL and ≥ 6mm PPD;  gingival bleeding in at least 8 teeth | ≥ 8 | **ST-segment elevation myocardial infarction (STEMI)**  typical chest pain at rest associated with ST-segment elevation of at least 1 mm in 2 contiguous leads in the frontal plane or 2 mm in the horizontal plane, or typical pain at rest in patients with a new, or presumably new, left bundle-branch block. |
| Wang et al. 2020 | Stages III/IV periodontitis | ≥ 6 sites with PD ≥ 4mm; >25% of interproximal sites with CAL ≥ 5mm; | ≥ 8 | **Type 2 diabetic**  HbA1c > 6.5% |
| Montero et al. 2020 | stages III-IV generalized periodontitis | ≥ eight sites with PPD ≥ 6 mm and four sites with CAL ≥ 5 mm, distributed in ≥ two different quadrants | ≥ 16 | **Metabolic syndrome (Met S)**  (1) elevated waist circumference (WC) (≥ 94 cm in men, ≥ 80 cm in women)  (2) elevated triglycerides (≥ 150 mg/dL),  reduced  (3) HDL cholesterol (< 40 mg/dL in males; < 50 mg/dL in females)  (3) elevated blood pressure (systolic ≥ 130 and/or diastolic ≥ 85 mm Hg),  (4) elevated fasting plasma glucose (FPG) (≥ 100 mg/dL), |
| Doke et al. 2021 | light–moderate periodontitis | 4 mm ≤ PD < 6 mm | NI | **Metabolic syndrome (Met S)**  abdominal obesity (men: ≥ 85 cm, women: ≥ 90 cm circumference at the navel), in addition to more than two of the following three statuses: diabetes (fasting blood sugar level: FBS level ≥ 110 mg/dl), hypertension (blood pressure ≥ 130/85 mmHg), dyslipidemia (triglyceride level: TG level ≥ 150 mg/dl and/or high density lipo-protein level: HDL level ≤ 40 mg/dl). |

CAL: Clinical Attachment Level; HbA1c: Glycated Haemoglobin; HDL: high-density lipoprotein; LDL: low-density lipoprotein; PD: probing depth; PPD: periodontal probing depth; NI: No Information; TG: Triglycerides.

**Supplemental Table S4: The intervention protocols of included studies**

| **Study** | **Test Intervention**  **(NSPT)** | | | **Control Intervention** | | **Follow-up** |
| --- | --- | --- | --- | --- | --- | --- |
|  | Teeth Extractions performed | At Baseline | At Follow-up | At Baseline | At Follow-up |  |
| Ide et al. 2003 | NI | OHI + Supra and Subgingival SRP | None | no treatment | | 6 weeks |
| D'Aiuto et al. 2005-1 | NI | subgingival mechanical instrumentation | None | no treatment | | 2 months |
| D'Aiuto et al. 2005-2 | NI | subgingival mechanical instrumentation + adjunctive local delivery of minocycline-HCl | None | no treatment | | 2 months |
| Tonetti et al. 2007 | Yes | OHI + Supra and Subgingival SRP + deliver microspheres of minocycline into the periodontal pockets. | None | OHI + supragingival mechanical scaling and polishing. | None | 6 months |
| Higashi et al. 2009 | NI | OHI + subgingival SRP + antibiotics were used for 4–7 days after therapy. | None | no treatment | | 24 weeks |
| Taylor et al. 2010 | Yes | OHI + SRP | None | no treatment | | 8 weeks |
| Li et al. 2011 | Yes | OHI + supra- and sub-gingival SRP + apply chlorhexidine gel into periodontal pockets with PD ≥ 4mm + Corsodyl 0.2% w/v Mint Mouthwash (chlorhexidine digluconate 0.2% w/v, 300 ml) for home use | None | OHI | None | 3 months |
| Kamil et al. 2011 | NI | OHI + SRP | After SRP, a professional plaque control program was performed twice a month during the follow-up period to reinforce the OHI and to rescale bleeding sites. | OHI | None | 3 months |
| Chen et al. 2012-1 | NI | OHI + SRP | At 3 months: OHI + Subgingival prophylaxis | No treatment | | 6 months |
| Chen et al. 2012-2 | NI | OHI + SRP | At 3 months: OHI + Supragingival prophylaxis | No treatment | | 6 months |
| Bokhari et al. 2012 | NI | OHI + supra- and sub-gingival SRP | None | no treatment | | 2 months |
| Wehmeyer et al. 2013 | Yes | OHI + supra- and subgingival microbial deposits by SRP | Adjunctive local delivery antimicrobial therapy with controlled-release microsphere encapsulated biodegradable minocycline was administered to all sites with > 5 mm probing depths at the time of scaling and root planning and at the 3-and 6-month follow-up appointments | OHI | None | 6 months |
| Kapellas et al. 2014 | NI | OHI + nonsurgical removal of sub- and supragingival calculus and plaque biofilm | None | OHI | None | 12 months |
| Fang et al. 2015 | Yes | OHI +supra- and sub-gingival mechanical instrumentation of the root surface (SRP) | At 3 months: supragingival prophylaxis | No treatment | | 3 months |
| Fu et al. 2016 | NI | OHI + SRP with ultrasonic devices and curettes + Tongue brushing with a 1 % chlorhexidine gel (1 min) + Mouth rinsing with a 0.2 % chlorhexidine solution (2 min) + Subgingival chlorhexidine (1 %) irrigation in all pockets + Twice daily rinsing with chlorhexidine (1 min) during 14 days after the periodontal intervention. | None | OHI | None | 6 months |
| Zhou et al. 2017) | Yes | OHI + removal of dental plaque biofilms with the use of SRP + minocycline hydrochloride ointment were delivered locally into the periodontal pockets once per week for four continued week | At 3 and 6 months: OHI + standard cycle of supragingival ultrasonic scaling and polishing if necessary | OHI + Supragingival scaling | At 3 and 6 months: OHI | 6 months |
| Staffi et al. 2018 | NI | OHI + supragingival plaque control + SRP | professional plaque biofilm removal and reinforcement of oral hygiene instructions once monthly | OHI + Supragingival scaling | None | 3 months |
| Montenegro et al. 2019 | NI | OHI + supragingival plaque control + SRP | Individual periodontal maintenance or recall visits (professional plaque removal and reinforcement of OHI) once monthly | OHI + removal of Supragingival plaque and calculus | None | 3 months |
| Czesnikiewicz-Guzik et al. 2019 | NI | OHI + subgingival and supragingival scaling | None | OHI + supragingival scaling | None | 2 months |
| Lobo et al. 2020 | Yes | OHI + subgingival and supragingival scaling | None | OHI | None | 6 months |
| Wang et al. 2020 | Yes | OHI + SRP | At 3 months: OHI + Plaque removal | OHI | At 3moths: OHI | 6 months |
| Montero et al. 2020 | NI | OHI + SRP + adjunctive administration of a systemic antibiotic (azithromycin 500 mg, q.d., for three days) | OHI | OHI + supragingival professional mechanical plaque removal + adjunctive administration of placebo medication (identical capsules containing lactose 500 mg, q.d, for 3 days) | OHI | 6 months |
| Doke et al. 2021 | NI | OHI + supra/subgingival SRP. | None | No treament | | 3 months |

NI: No Information; NSPT: Non-Surgical Periodontal Therapy; OHI: oral hygiene instructions; SRP: scaling and root planning.

**Supplemental Table S5****: Subgroup analysis according to systemic health status.**

|  | Systemic health status | No. study | *I^2^*(%) | WMD[95% CI] | *P* for intra-group | *P* for between groups |
| --- | --- | --- | --- | --- | --- | --- |
| CRP | otherwise healthy | 6 | 54 | -0.32[-0.93; 0.28] | 0.29 | 0.02 |
|  | CVD | 3 | 0 | -1.20[-1.26; -1.14] | < 0.01 |  |
|  | metabolic disorders | 4 | 0 | -0.91[-1.77; -0.05] | 0.04 |  |
| IL6 | CVD | 3 | 0 | -1.26[-3.25; 0.73] | 0.21 | 0.34 |
|  | metabolic disorders | 3 | 5 | -0.65[-0.82; -0.48] | < 0.01 |  |
|  | ESRD | 2 | 0 | -1.47[-2.46; -0.48] | < 0.01 |  |
| IL-1 | CVD | 2 | 0 | -1.33[-3.47; 0.80] | 0.22 | 0.33 |
|  | metabolic disorders | 2 | 92 | -0.16[-1.20; 0.88] | 0.76 |  |
| TNF | CVD | 2 | 0 | -0.37[-2.62; 1.88] | 0.75 | 0.44 |
|  | metabolic disorders | 4 | 48 | -1.49[-3.20; 0.23] | 0.09 |  |
| LDL | otherwise healthy | 3 | 47 | -0.21[-0.45; 0.02] | 0.08 | 0.46 |
|  | CVD | 2 | 0 | 0.03[-0.29; 0.35] | 0.87 |  |
|  | metabolic disorders | 5 | 0 | -0.18 [-0.37; 0.01] | 0.06 |  |
| HDL | otherwise healthy | 3 | 0 | -0.07[-0.20; 0.06] | 0.28 | 0.07 |
|  | CVD | 2 | 0 | 0.01[-0.09; 0.12] | 0.79 |  |
|  | metabolic disorders | 5 | 0 | 0.09[0.02; 0.15] | < 0.01 |  |
| TC | otherwise healthy | 3 | 0 | 0.02[-0.26; 0.29] | 0.91 | 0.76 |
|  | CVD | 2 | 0 | 0.12[-0.25; 0.48] | 0.52 |  |
|  | metabolic disorders | 4 | 4.5 | -0.05[-0.29; 0.19] | 0.70 |  |
| TG | otherwise healthy | 3 | 0 | 0.03[-0.18; 0.24] | 0.77 | 0.11 |
|  | CVD | 2 | 0 | 0.04[-0.32; 0.40] | 0.83 |  |
|  | metabolic disorders | 5 | 71.0 | -0.24[-0.43; -0.06] | 0.01 |  |
| SBP | CVD | 2 | 49.6 | -6.28[-14.82; 2.25] | 0.15 | 0.93 |
|  | metabolic disorders | 3 | 72.5 | -6.92[-17.02; 3.18] | 0.18 |  |
| DBP | CVD | 2 | 0 | -4.51[ -7.82; -1.20] | < 0.01 | 0.73 |
|  | metabolic disorders | 3 | 83.1 | -2.81[-11.71; 6.10] | 0.54 |  |

**Supplemental Table S6: Subgroup analysis according to usage of antiseptic/antibiotics.**

|  | Use of antiseptic/antibiotics | No. study | *I^2^*(%) | WMD [95% CI] | *P* for intra-group | *P* for between groups |
| --- | --- | --- | --- | --- | --- | --- |
| CRP | yes | 6 | 53.4 | -0.41 [-1.11; 0.29] | 0.25 | 0.47 |
|  | no | 8 | 67.2 | -0.74 [-1.26; -0.22] | < 0.01 |  |
| IL6 | yes | 6 | 0 | -0.66 [-0.83; -0.49] | < 0.01 | 0.01 |
|  | no | 4 | 0 | -1.42 [-1.96; -0.89] | < 0.01 |  |
| IL-1 | yes | 2 | 92.0 | -0.16 [-1.20; 0.88] | 0.76 | 0.81 |
|  | no | 3 | 2.1 | -0.34 [-1.29; 0.62] | 0.49 |  |
| TNF | yes | 3 | 51.0 | -1.68 [-4.44; 1.07] | 0.23 | 0.25 |
|  | no | 5 | 0 | -0.06 [-0.50; 0.38] | 0.79 |  |
| LDL | yes | 4 | 0 | -0.21 [-0.41; -0.02] | 0.03 | 0.16 |
|  | no | 8 | 11.1 | -0.04 [-0.18; 0.09] | 0.53 |  |
| HDL | yes | 4 | 6.0 | 0.09 [0.02; 0.16] | 0.01 | 0.09 |
|  | no | 9 | 0 | 0.01 [-0.04; 0.07] | 0.66 |  |
| TC | yes | 4 | 2.2 | -0.00 [-0.24; 0.23] | 0.99 | 0.57 |
|  | no | 8 | 0 | 0.08 [-0.07; 0.23] | 0.30 |  |
| TG | yes | 4 | 76.7 | -0.22 [-0.39; -0.05] | 0.01 | 0.03 |
|  | no | 7 | 0 | 0.04 [-0.12; 0.19] | 0.63 |  |
| SBP | yes | 4 | 46.5 | -11.40 [-13.33; -9.46] | < 0.01 | < 0.01 |
|  | no | 2 | 0 | -1.97 [-8.00; 4.05] | 0.52 |  |
| DBP | yes | 4 | 57.9 | -7.24 [-10.82; -3.66] | < 0.01 | < 0.01 |
|  | no | 2 | 0 | 1.00 [-2.44; 4.44] | 0.57 |  |

**Supplemental Table S7: Subgroup analysis according to follow-up time.**

|  | Follow-up time | No. study | *I^2^*(%) | WMD [95% CI] | *P* for intra-group | *P* for between groups |
| --- | --- | --- | --- | --- | --- | --- |
| CRP | <6 months | 6 | 73.8 | -0.64 [-1.21; -0.07] | 0.03 | 0.88 |
|  | ≥6 months | 8 | 47.8 | -0.57 [-1.20; 0.06] | 0.08 |  |
| IL6 | <6 months | 2 | 0 | -5.54 [-12.67; 1.59] | 0.13 | 0.19 |
|  | ≥6 months | 8 | 37.0 | -0.73 [-0.89; -0.57] | < 0.01 |  |
| IL-1 | <6 months | 3 | 2.1 | -0.34 [-1.29; 0.62] | 0.49 | 0.81 |
|  | ≥6 months | 2 | 92.0 | -0.16 [-1.20; 0.88] | 0.76 |  |
| TNF | <6 months | 3 | 0 | 0.17 [-0.15; 0.48] | 0.29 | 0.08 |
|  | ≥6 months | 5 | 62.5 | -1.08 [-2.42; 0.26] | 0.11 |  |
| LDL | <6 months | 6 | 31.0 | -0.09 [-0.25; 0.07] | 0.25 | 0.94 |
|  | ≥6 months | 6 | 0 | -0.10 [-0.26; 0.05] | 0.20 |  |
| HDL | <6 months | 6 | 0 | 0.00 [-0.07:0.07] | 0.93 | 0.19 |
|  | ≥6 months | 7 | 0 | 0.06 [0.01;0.12] | 0.02 |  |
| TC | <6 months | 5 | 0 | 0.1 [-0.09; 0.29] | 0.31 | 0.55 |
|  | ≥6 months | 7 | 0 | 0.02 [-0.14; 0.19] | 0.78 |  |
| TG | <6 months | 5 | 0 | 0.06 [-0.15; 0.26] | 0.59 | 0.12 |
|  | ≥6 months | 6 | 68.3 | -0.14 [-0.28; -0.00] | 0.05 |  |
| SBP | <6 months | 2 | 0 | -7.86 [-12.81; -2.91] | < 0.01 | 0.99 |
|  | ≥6 months | 4 | 75.7 | -7.82 [-15.76; 0.12] | 0.05 |  |
| DBP | <6 months | 2 | 82.8 | -5.67 [-11.38;0.05] | 0.74 | 0.41 |
|  | ≥6 months | 4 | 81.5 | -1.44 [ -9.77; 6.90] | 0.05 |  |

CI: confidence interval; CRP: C-reactive protein, CVD: Cardiovascular disease, DBP: diastolic blood pressure, HDL: high-density lipoprotein, IL-1β: Interleukin-1β, IL-6: Interleukin-6, LDL; low-density lipoprotein, SBP: Systolic Blood pressure, TC: total cholesterol, TG: triglycerides. TNF-α: tumor necrosis factor, WMD: weighted mean differences.
