# Supplementary material for: Effect of non-surgical periodontal therapy on risk markers of cardiovascular disease: a systematic review and meta-analysis
Source: BMC Oral Health. 2024 Jun 14;24:692. doi: 10.1186/s12903-024-04433-0 (PMC11177403; doi:10.1186/s12903-024-04433-0)
Supplement: Supplementary file 2 — Supplementary Material 2 [file 12903_2024_4433_MOESM2_ESM.docx]

**List of Supplemental Figure**

Fig. S1. Sensitivity analysis for the levels of systemic inflammation markers by excluding studies one by one. (A)CRP; (B)IL-6; (C)IL-1β; (D)TNF-α.

Fig. S2. Sensitivity analysis for the levels of lipids by excluding studies one by one. (A)LDL; (B)HDL; (C)TC; (D)TG.

Fig. S3. Sensitivity analysis for vascular function by excluding studies one by one. (A)SBP; (B)DBP.

Fig. S4. Sensitivity analysis for the levels of systemic inflammation markers by excluding the studies in which the control group performed CPT. (A)CRP; (B)IL-6; (C)TNF-α.

Fig. S5. Sensitivity analysis for the levels of lipids by excluding the studies in which the control group performed CPT. (A)LDL; (B)HDL; (C)TC; (D)TG.

Fig. S6. Sensitivity analysis for vascular function by excluding the studies in which the control group performed CPT. (A)SBP; (B)DBP.

Fig. S7. Funnel plot and Egger’s test of (A) CRP, (B)IL-6, (C)LDL, (D)HDL, (E)TC, (F)TG.


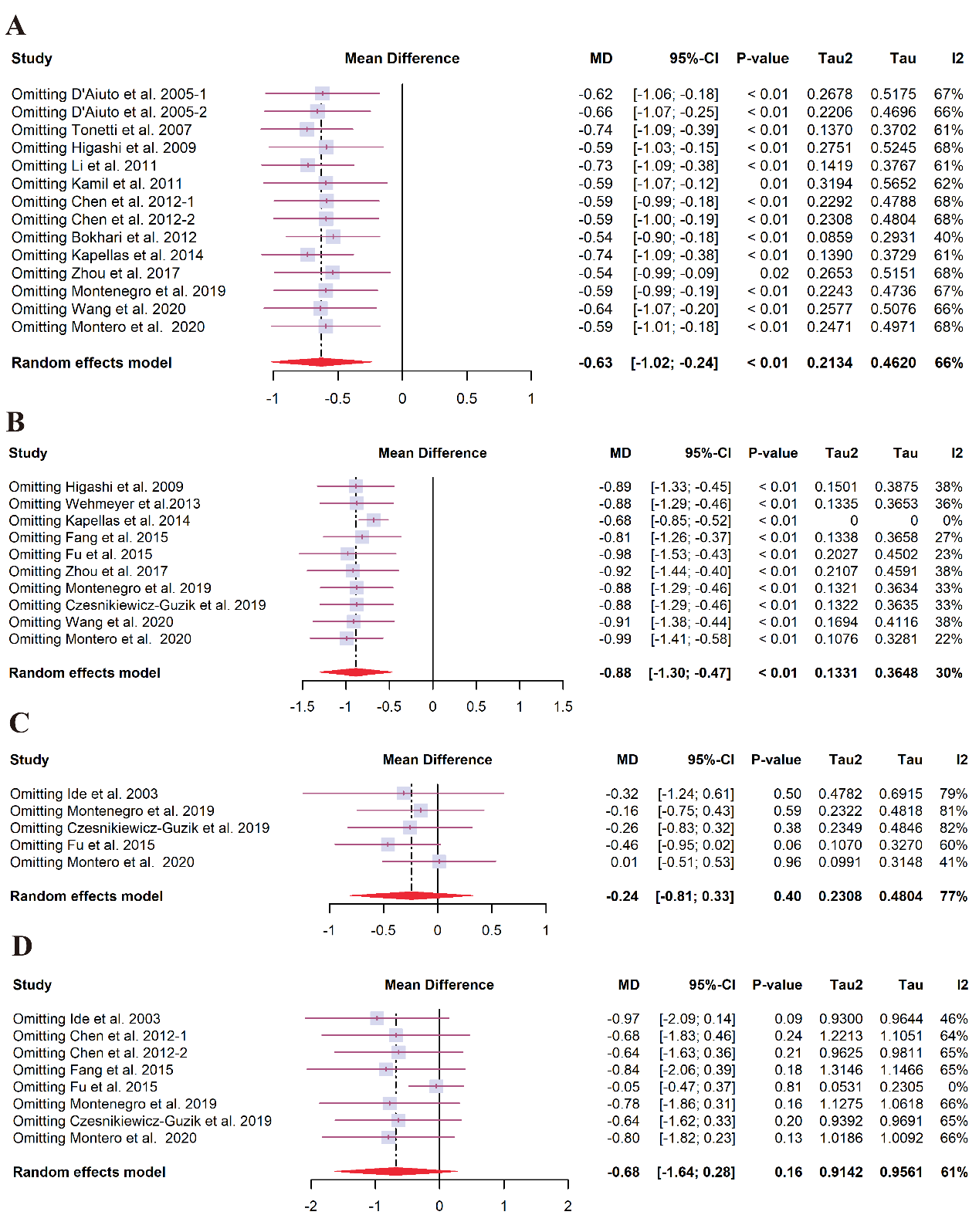
Fig. S1. Sensitivity analysis for the levels of systemic inflammation markers by excluding studies one by one. (A)CRP; (B)IL-6; (C)IL-1β; (D)TNF-α.


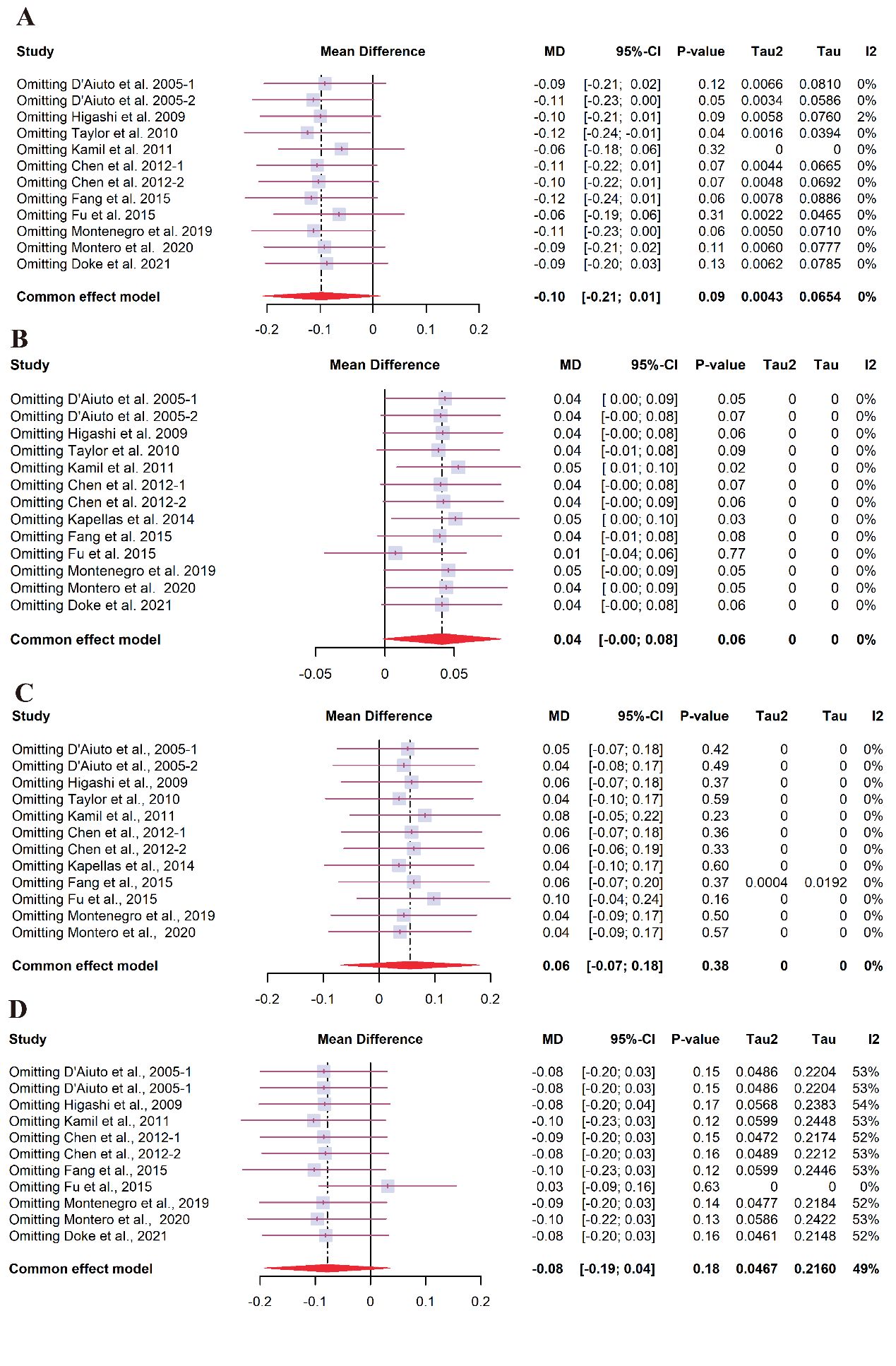


Fig. S2. Sensitivity analysis for the levels of lipids by excluding studies one by one. (A)LDL;(B)HDL; (c)TC; (D)TG.


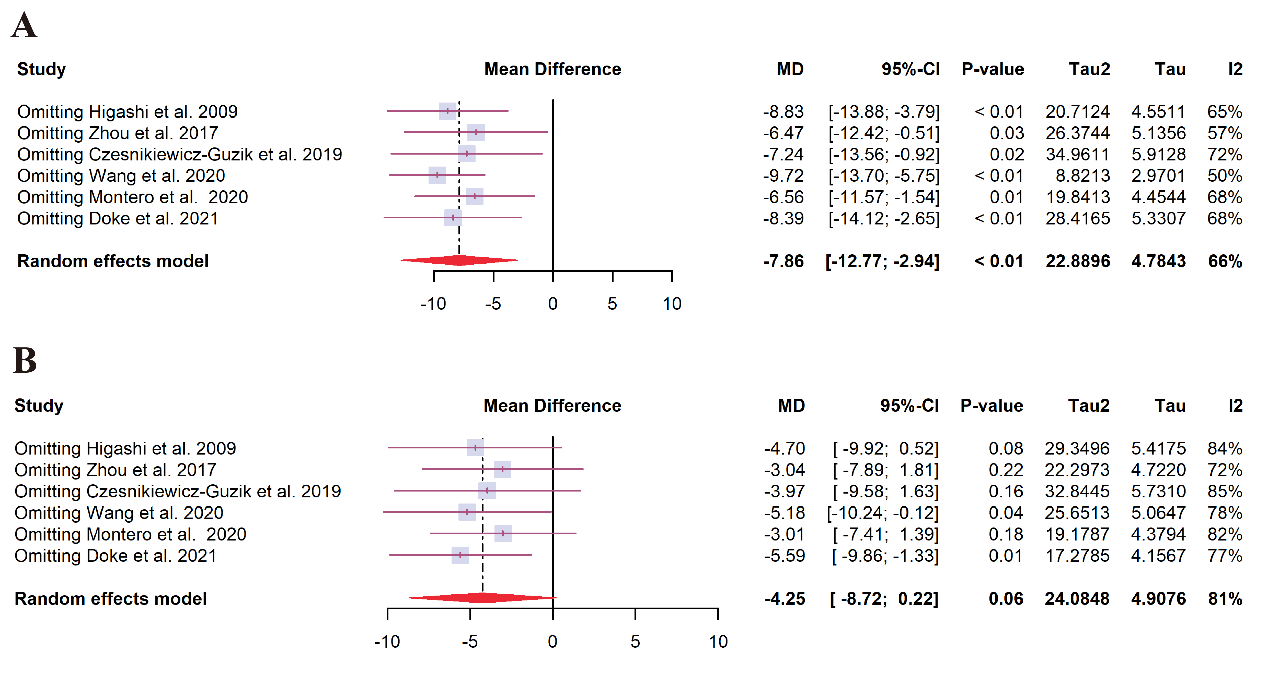


Fig. S3. Sensitivity analysis for vascular function by excluding studies one by one. (A)SBP; (B)DBP.


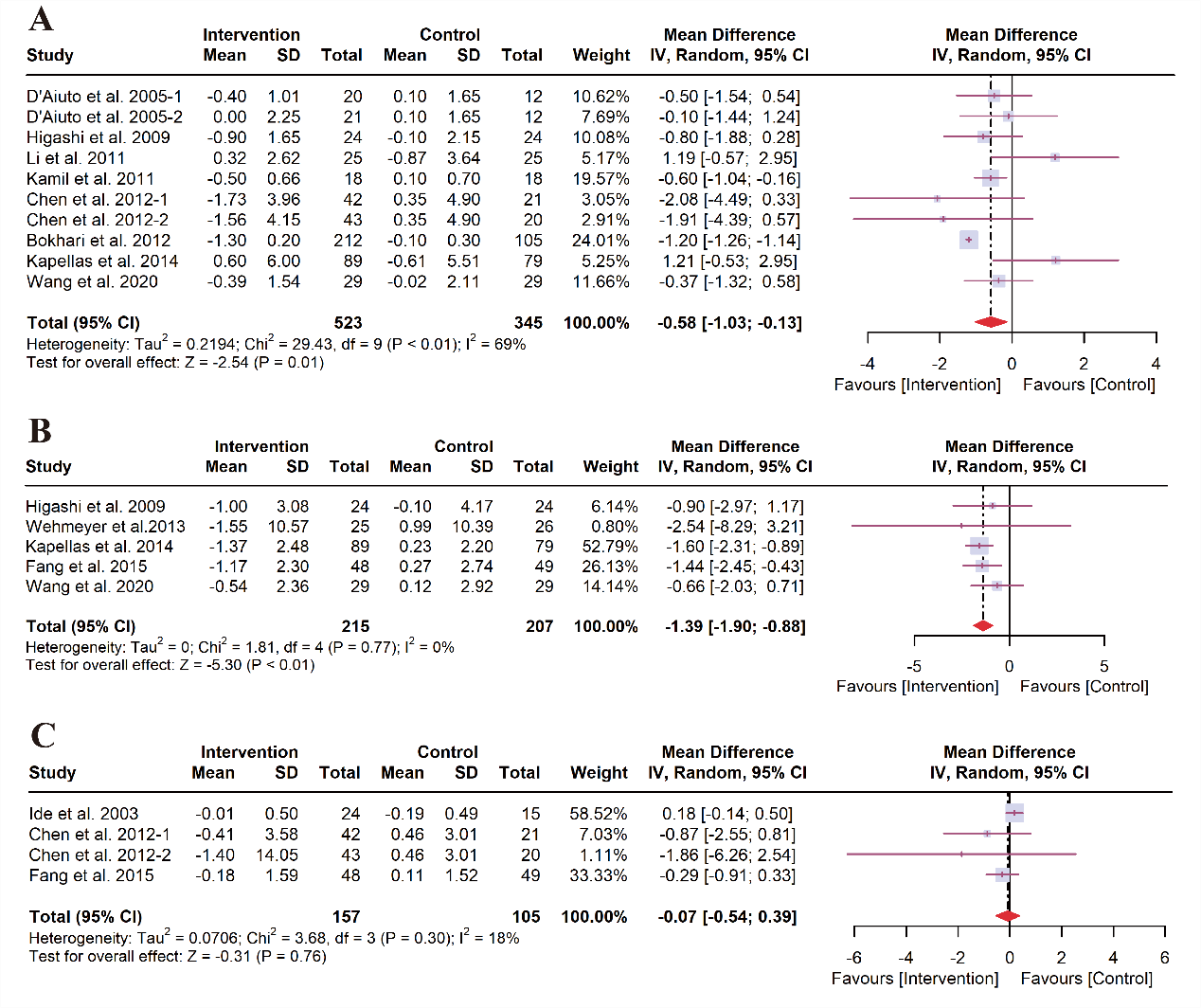
Fig. S4. Sensitivity analysis for the levels of systemic inflammation by excluding the studies in which the control group performed CPT. (A)CRP; (B)IL-6; (C)TNF-α.


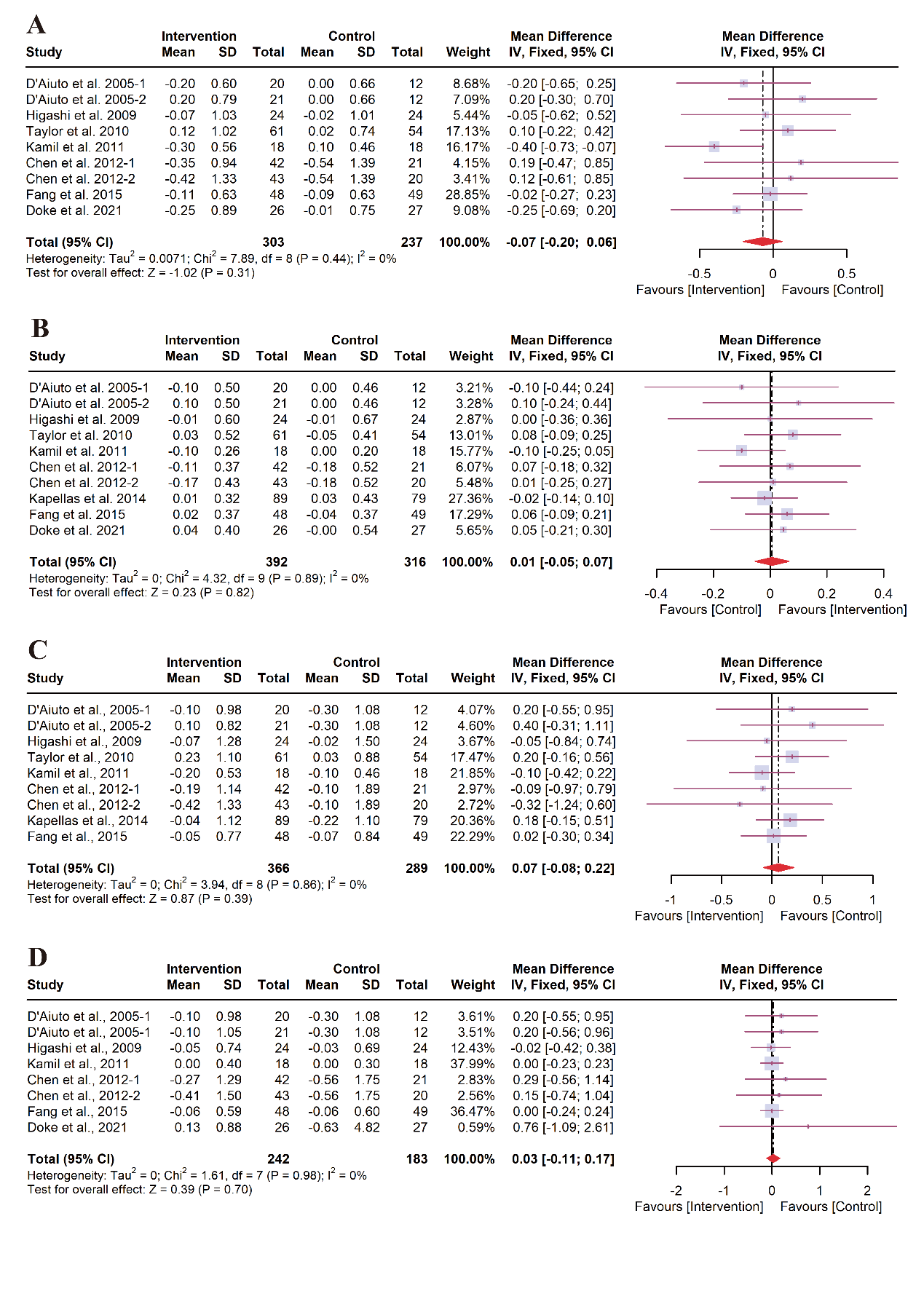
Fig. S5. Sensitivity analysis for the levels of lipids by excluding the studies in which the control group performed CPT. (A)LDL; (B)HDL; (C)TC; (D)TG.


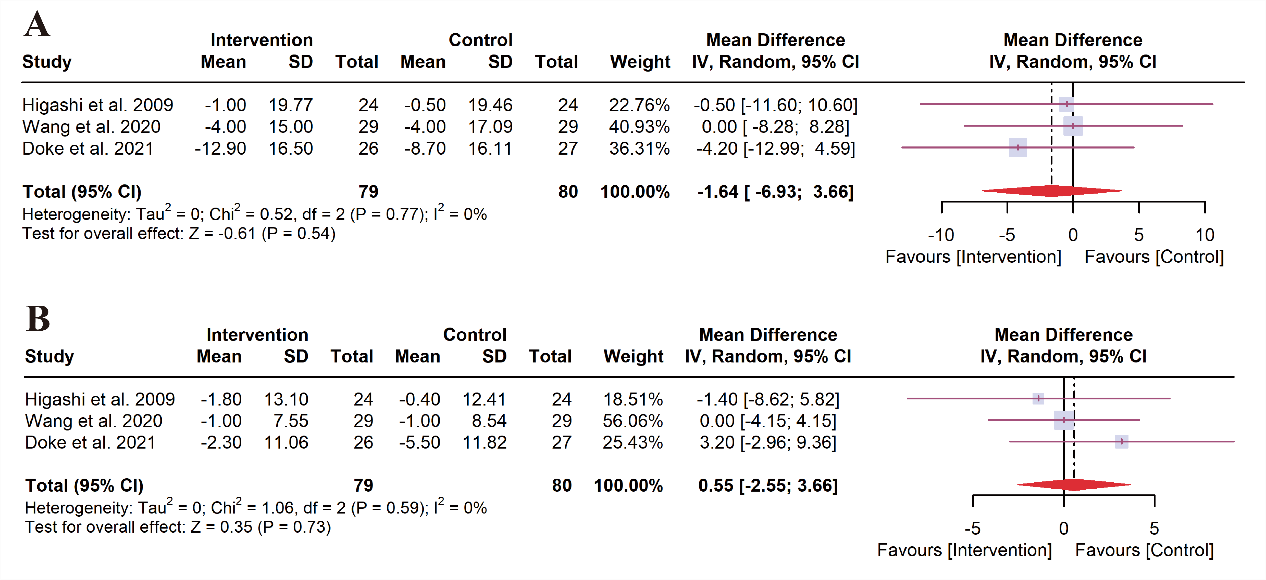


Fig. S6. Sensitivity analysis for vascular function by excluding the studies in which the control group performed CPT. (A)SBP; (B)DBP.


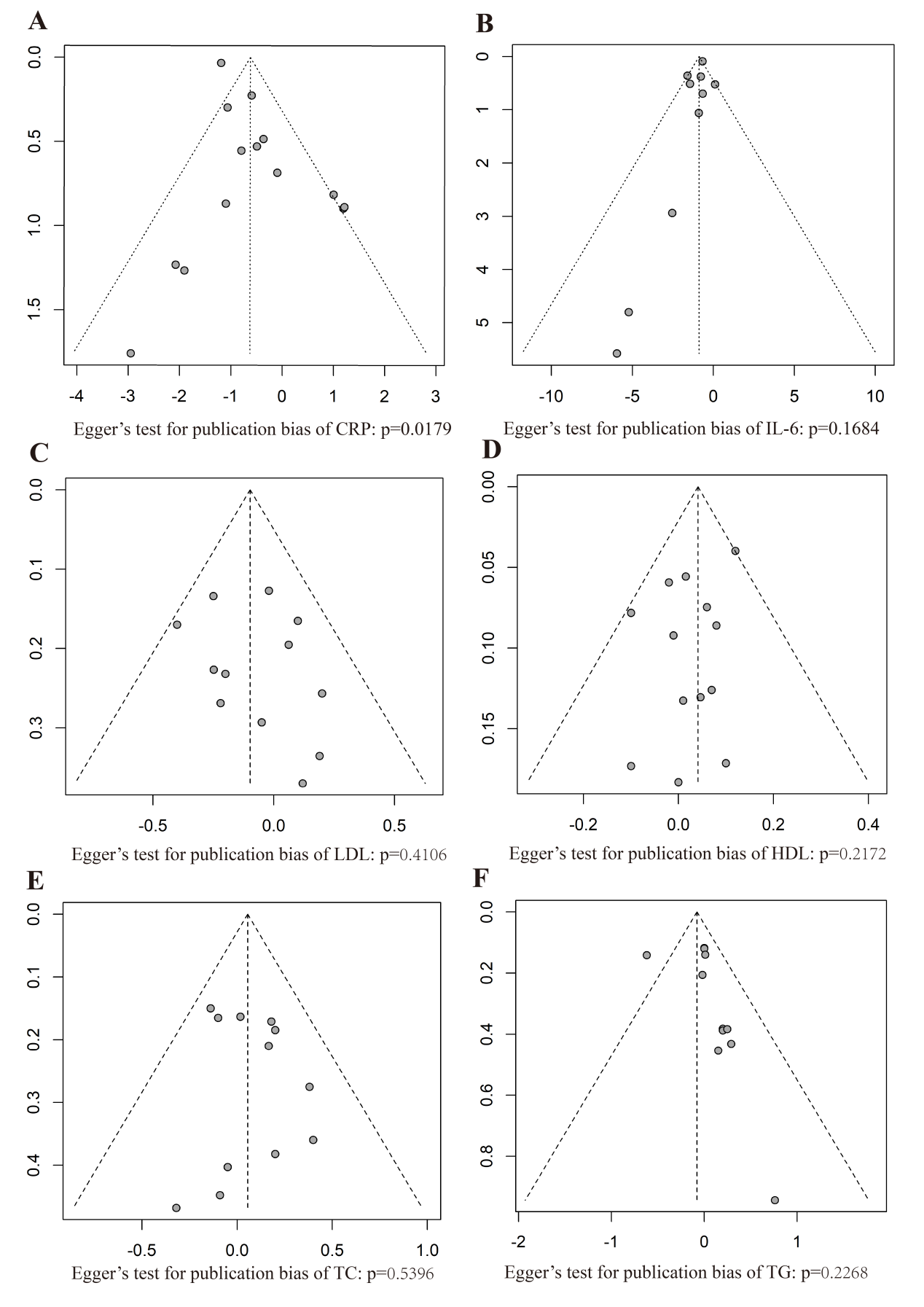
Fig. S7. Funnel plot and Egger’s test of (A) CRP, (B)IL-6, (C)LDL, (D)HDL, (E)TC, (F)TG.
